# Impact of HCV *core* gene quasispecies on hepatocellular carcinoma risk among HALT-C trial patients

**DOI:** 10.1038/srep27025

**Published:** 2016-06-01

**Authors:** Ahmed El-Shamy, Matthew Pendleton, Francis J. Eng, Erin H. Doyle, Ali Bashir, Andrea D. Branch

**Affiliations:** 1 Division of Liver Diseases, Department of Medicine, Icahn School of Medicine at Mount Sinai, New York, NY, USA; 2 Department of Genetics and Genomic Sciences, Icahn School of Medicine at Mount Sinai, New York, NY, USA

## Abstract

Mutations at positions 70 and/or 91 in the core protein of genotype-1b, hepatitis C virus (HCV) are associated with hepatocellular carcinoma (HCC) risk in Asian patients. To evaluate this in a US population, the relationship between the percentage of 70 and/or 91 mutant HCV quasispecies in baseline serum samples of chronic HCV patients from the HALT-C trial and the incidence of HCC was determined by deep sequencing. Quasispecies percentage cut-points, ≥42% of non-arginine at 70 (non-R^70^) or ≥98.5% of non-leucine at 91 (non-L^91^) had optimal sensitivity at discerning higher or lower HCC risk. In baseline samples, 88.5% of chronic HCV patients who later developed HCC and 68.8% of matched HCC-free control patients had ≥42% non-R^70^ quasispecies (*P* = 0.06). Furthermore, 30.8% of patients who developed HCC and 54.7% of matched HCC-free patients had quasispecies with ≥98.5% non-L^91^ (*P* = 0.06). By Kaplan-Meier analysis, HCC incidence was higher, but not statistically significant, among patients with quasispecies ≥42% non-R^70^ (*P* = 0.08), while HCC incidence was significantly reduced among patients with quasispecies ≥98.5% non-L^91^ (*P* = 0.01). In a Cox regression model, non-R^70^ ≥42% was associated with increased HCC risk. This study of US patients indicates the potential utility of HCV quasispecies analysis as a non-invasive biomarker of HCC risk.

Patients with chronic hepatitis C virus (HCV) infection have an increased risk of developing hepatocellular carcinoma (HCC)^1^. The risk is particularly high for patients with advanced fibrosis/cirrhosis^2^. Virologic cure, as indicated by a sustained virological response (SVR) after completion of antiviral therapy, does not eliminate the risk of developing HCC^3^. Patients with advanced fibrosis/cirrhosis are advised to undergo bi-annual HCC screening^4^,^5^. Early detection of HCC improves outcome^2^. A better understanding of hepatocellular carcinogenesis and a greater ability to identify high-risk patients would allow surveillance and prevention efforts to be focused on the individuals at highest HCC risk who are most likely to benefit.

Host risk factors for HCV-associated HCC include advanced fibrosis/cirrhosis, older age at time of infection, male sex, coinfection with human immunodeficiency virus (HIV) or hepatitis B virus (HBV), diabetes, obesity and prolonged heavy-use of alcohol^6^,^7^. Among patients infected with genotype-1b, those infected with a virus harboring amino acid substitutions at position 70 and/or 91 in the core protein have an elevated HCC risk^8^. The close association between amino acid 70 substitutions and increased HCC risk has been observed in several independent patient cohorts, but primarily in Asian (Japanese) populations^9^,^10^,^11^,^12^,^13^,^14^,^15^,^16^. Most notably, the increased HCC risk associated with amino acid 70 substitutions persists even after successful eradication of the virus with antiviral therapy^17^. Experimental studies in mice and in cell culture have confirmed the oncogenic potential of the HCV *core* gene^18^,^19^,^20^. However, the association of amino acid substitutions in the core protein with an increased risk of developing HCC has not previously been examined in a non-Asian population of HCV-infected patients.

In addition, due to the error-prone replication machinery of HCV and evolutionary pressure from the host immune system, mutant viral sequences arise and accumulate in each patient, creating a genetically varied HCV RNA population. The swarm of sequence variants is called a quasispecies. We sought to determine whether the percentage of the quasispecies with mutant amino acids at positions 70 and/or 91 at baseline was associated with the incidence of HCC. The advent in recent years of next generation deep sequencing methods allowed this question to be addressed. Akuta and colleagues recently used this type of approach to determine the impact of these positions on the risk of developing HCC after achieving an SVR^17^. They found that patients whose pre-treatment viral quasispecies contained ≥20% mutant sequences at position 70 had an increased incidence of HCC post-SVR. Mutant sequences were defined as those not coding for arginine at position 70 (non-R^70^). Although there are strong data from Japan about the importance of core protein mutations, it was not known whether they are important in a non-Asian population.

This study was undertaken to investigate the significance of *core* gene mutations at codons 70 and 91 in a non-Asian population of patients with genotype-1b HCV and to evaluate the relationship between HCC risk and the percentage of the quasispecies with mutant sequences for codons 70 and 91. Serum samples and clinical data were obtained from the archives of the Hepatitis C Antiviral Long-term Treatment against Cirrhosis (HALT-C) trial. The HALT-C trial was a prospective multi-center, randomized, controlled trial that evaluated the impact of long-term half-dose pegylated interferon (PEG-IFN) therapy on clinical and histologic progression of chronic hepatitis in patients with advanced fibrosis who had previously failed to respond to antiviral therapy^21^. These patients were ideal because baseline samples were available from the time of enrollment and patients were followed for up to 8.5 years to determine the incidence of HCC^22^. Our hypothesis is that the HCV core protein is indeed a viral factor that promotes oncogenesis and that amino acid substitutions in the core protein of genotype-1b HCV may further enhance oncogenesis independent of the country of origin of the patient or the virus. Identification of *core* gene mutations by analyzing HCV RNA from blood may serve as a non-invasive indicator of HCC risk.

## Patients and Methods

### HALT-C trial design

Two groups of patients with chronic HCV infection who failed PEG-IFN/ribavirin (RBV) therapy were enrolled into the long-term phase of the HALT-C trial. One group was comprised of patients who failed PEG-IFN/ribavirin (RBV) therapy administered during a lead-in phase of the trial; those with detectable HCV RNA at 20 weeks of PEG-IFN/RBV were randomized at week 24 to receive either half dose of PEG-IFN (RBV was discontinued) or no treatment for the following 3.5 years. The other “express” group was comprised of patients who failed therapy outside the trial. These patients were randomized directly to either the low-dose PEG-IFN arm or to the no treatment arm of the long-term phase. None of the patients achieved an SVR during the long-term phase; all continued to have chronic HCV infection. All patients in the long-term phase were followed for a median of 6.7 years and a maximum of 8.5 years. Patients were seen every 3 months during the first 3.5 years of the long-term phase and every 6 months thereafter. Patients underwent hepatic ultrasound examination every 6–12 months to screen for HCC. Patients with an elevated or rising AFP and those with new lesions on ultrasound were further evaluated with CT or MRI. HCC diagnosis was based on histological confirmation or imaging with or without AFP levels increasing to >1000 ng/mL. Control patients (non-HCC) were defined as patients who were not diagnosed with HCC up to the last follow up visit. The HALT-C trial was registered in ClinicalTrials.gov number: NCT00006164 and it was registered on August 8, 2000 (https://clinicaltrials.gov/ct2/show/NCT00006164). The entire HALT-C study protocol is provided in the supplementary file. The protocol was carried out in accordance with the approval of Institutional Review Board (IRB) committees at the following institutions:

University of Massachusetts Medical Center, Worcester; University of Connecticut Health Center, Farmington; Saint Louis University School of Medicine, St. Louis; Massachusetts General Hospital, Boston; University of Colorado School of Medicine, Denver; University of California, Irvine; University of Texas Southwestern Medical Center, Dallas; University of Southern California, Los Angeles; University of Michigan Medical Center, Ann Arbor; Virginia Commonwealth University Health System, Richmond; Liver Diseases Branch, National Institute of Diabetes and Digestive and Kidney Diseases, National Institutes of Health, Bethesda, MD; University of Washington, Seattle; Central Virology Laboratory, New England Research Institutes, Watertown, MA; Data Coordinating Center and Armed Forces Institute of Pathology, Washington, DC – Central Pathology Reading Center. All patients provided written informed consent for participation in the trial.

### HALT-C study group

Our study group only included individuals in the HALT-C trial with genotype-1b HCV who developed HCC (n = 26) and matched controls who did not develop HCC (n = 64). HCC (cases) and controls were matched for baseline histopathology (fibrosis/cirrhosis) and for assignment to one of the two arms of the HALT-C trial. They were also matched on length of follow-up (controls had to be followed longer than cases). Baseline serum samples were those collected just prior to assignment to one of the two arms in the long-term phase of the HALT-C trial, after patients failed to achieve an SVR with interferon-based HCV treatment. The sub-genotype was determined by Sanger sequencing of the 5′ UTR and the *core* gene.

### Amplification and sequence analysis of mutations in the core gene

RNA was isolated from 200 μL of serum using QIAamp Viral RNA kit (Qiagen, Hilden, Germany) according to manufacturer’s instructions. RNA was reverse transcribed (RT) using SuperScript III First-Strand system (Life Technologies, Gaithersburg, MD) and an HCV-specific reverse-primer; E1 IN Reverse (5′ GTTCATCATCATATCCCATGCCAT 3′, HCV nt. 1281–1304). The RT reaction was performed at 65 °C for 30 minutes. The cDNA was amplified in a 35 cycle, single round polymerase chain reaction (PCR) using Phusion Hot Start II High-Fidelity DNA Polymerase (Thermo Fisher Scientific, Waltham, MA). Primers used for cDNA amplification were E1 IN Reverse and HCV primer RC1 (5′ GTCTAGCCATGGCGTTAGTA 3′, HCV nt. 65–84). Each PCR cycle consisted of denaturation at 98 °C for 10 seconds, annealing at 57 °C for 30 seconds and extension at 72 °C for 40 seconds. Amplicons were subsequently sequenced by Illumina-MiSeq deep-sequencing method following standard protocol (Illumina, San Diego, CA)^23^.

### Analysis of Illumina-MiSeq data

A workflow for the MiSeq analysis is shown in Supplemental Fig. 1. MiSeq reads were quality filtered, trimmed, and barcode filtered using Casava version 1.8 (Illumina, San Diego, CA). Given the high-depth of coverage per sample, only barcodes with zero or one mismatches were analyzed. After partitioning reads by sample barcodes, FASTA files were aligned to the HCV Con1 reference genome (accession no. AJ238799). Illumina reads were aligned using BWA-MEM version 0.7.4^24^ in paired-end mode with default parameters, and read pairs were required to map uniquely. After alignment, variants were generated at each reference position. For MiSeq data, the ‘mpileup’ option from SAMtools was used^25^. After pileup, the frequency of aligned bases at each reference position was recorded. Reported analyses are limited to single nucleotide variations (SNVs) in codons 70 and 91 of the HCV *core* gene. The association between core “haplotypes” (pairs of amino acids at positions 70 and 91 located on the same viral RNA) with HCC development was also analyzed. MiSeq reads were aligned using BWA-MEM (as described previously), and only reads which contained both positions 70 and 91 were selected.

### Power calculation

One of the objectives of the study was to generate the sequencing data needed to determine the sample size necessary to definitively test the hypothesis that HCV *core* gene mutations influence HCC risk. A mutant sequence was defined as having a codon that encoded an amino acid other than arginine at position 70 (non-R^70^) or leucine at position 91 (non-L^91^). Isolatess were to be classified as non-wild type if ≥50% of the quasispecies encoded a non-R^70^ or non-L^91^ codon. At the time the study was designed, the number of HALT-C patients with genotype-1b HCV who developed HCC during follow up was not known, but was projected to be 45 and the number of control (non-HCC) samples available for matching was anticipated to be at least 135. With this sample size, the study had a 63% power to detect a difference between the percentage of patients in whom ≥50% of the quasispecies encoded non-R^70^ between the two groups, assuming rates of 35% for controls and 56% for cases, as determined by using a two-tailed chi square test at a 5% confidence level.

### Statistical analysis

Statistical differences in the baseline parameters of HCC and non-HCC groups were determined by Student’s *t* test for numerical variables and Fisher’s exact probability test for categorical variables. Likewise, statistical differences in viral mutations between HCC and non-HCC groups were determined by Fisher’s exact probability test. The relationship between the incidence of HCC and the percentage of these non-R^70^ sequences present within the quasispecies was evaluated by receiver operating characteristic (ROC) analysis. Our study was designed to have three controls for each case, but genotyping of the samples revealed that fewer patients than expected had genotype-1b HCV infection. As a result, not all of the cases had three controls. Therefore, we performed both unmatched Cox regression and matched conditional logistic regression multivariable analyses to identify variables that independently associate with HCC development. Variables with a *P* <0.1 in univariable analysis were included in a backward stepwise multivariable Cox regression or matched conditional logistic analysis. Kaplan-Meier analysis was performed to estimate the cumulative incidence of HCC. A *P* < 0.05 was considered statistically significant. The association between core haplotypes and HCC was analyzed by ROC and Fisher’s exact probability test.

## Results

### Baseline characteristics of the study subjects

Baseline characteristic of patients with and without incident HCC were compared to each other (Table 1). At baseline, the group who later developed HCC (n = 26) (median follow up of 4.4 years; range of 0.4 to 8.5 years) had significantly lower white blood cell counts, platelet counts and albumin levels than the group who remained HCC-free (n = 64) during a median follow up of 7 years (range of 2.7 to 8.6 years). The group who developed HCC had significantly higher AST/ALT ratios, levels of alkaline phosphatase, and AFP at baseline. Fibrosis score (Ishak 5 or 6) and assignment to one of the two arms of the HALT-C trial (IFN/no-treatment) were matching variables and therefore would not be significant by design.

### Quasispecies at position 70 and HCC risk

**Figure 1:**
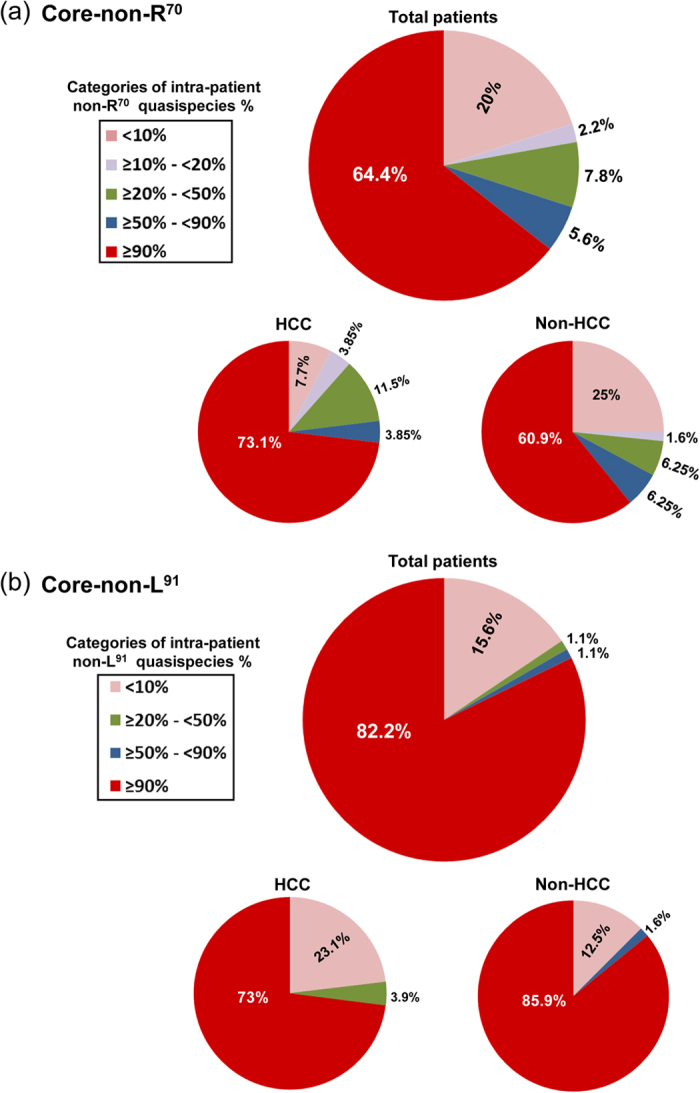
Frequency of intra-patient non-R^70^ and non-L^91^ quasispecies at various percentage cut-points in baseline samples among total patients (n = 90), those who later developed HCC (n = 26) and those who did not (non-HCC) (n = 64). The non-R^70^ (**a**) and non-L^91^ (**b**) intra-patient quasispecies cut-point percentages are categorized and indicated in the box legends.

**Figure 2:**
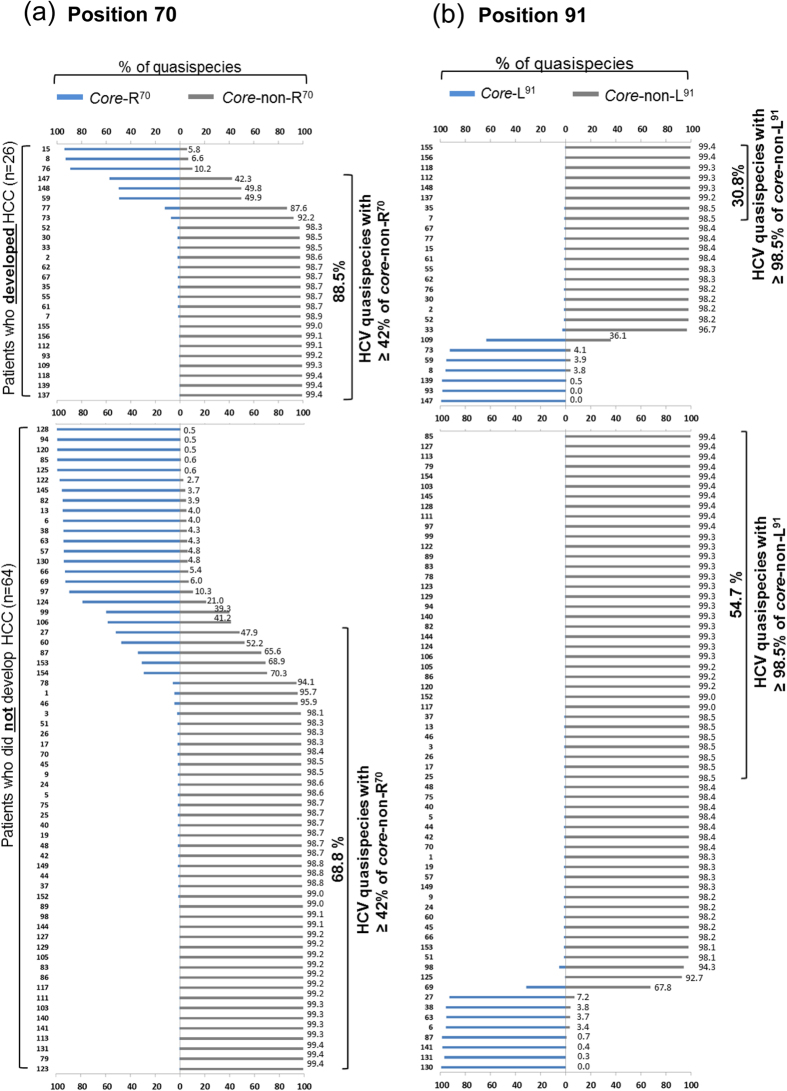
Proportion of baseline HCV core quasispecies at positions 70 (**a**) and 91(**b**) among patients who later developed HCC (n = 26) and patients who did not develop HCC (n = 64) as determined with Illumina-MiSeq data. Top and bottom axes of each panel indicate the percentage of quasispecies for core-R^70^, core-non-R^70^, core-L^91^ and core-non-L^91^. The exact percentage of core-non-R^70^ and core-non-L^91^ quasispecies are indicted at the end of each bar. Baseline samples containing quasispecies cut-points at ≥42% for core-non-R^70^ or at ≥98.5% for core-non-L^91^ are bracketed on the right side of each panel and indicate their frequency within HCC or non-HCC patients.

Illumina-MiSeq sequencing yielded a mean of 390,422 ± 145,407 and 297,789 ± 101,275 reads per sample of the HCC and non-HCC groups, respectively. The quasispecies of all patients (HCC and non-HCC) contained a mixture of R^70^ and non-R^70^ variants (Figs 1a and 2a). Although arginine (R) is the most common amino acid at position 70 in most genotype-1b sequences^26^, arginine was not the most common amino acid at position 70 in the quasispecies of this series. The high prevalence of mutant amino acids may be due to the positive selection of non-R^70^ with interferon treatment, as previously demonstrated by Kurbanov *et al.*^27^. The baseline samples analyzed in our study were obtained after PEG-IFN/RBV treatment failure, at the time of entry into the long-term phase of the HALT-C trial. Furthermore, the presence of non-R^70^ amino acids before IFN treatment has been shown to be strongly associated with IFN treatment failure^28^, and since our entire study group had failed IFN treatment, it is not surprising that non-R^70^ is highly represented in this patient group.

The relationship between the incidence of HCC and the percentage of non-R^70^ sequences in the quasispecies was evaluated using a range of percentage cut-points (≥10, 20, 40, 50, and 90%) (Table 2). Of the cut-points tested, ≥10% non-R^70^ had the lowest *P* value (0.08), while ≥50%, the pre-determined cut-point of the study, had the highest *P* value (0.3). The cut-point with the greatest ability to discriminate between patients who did or did not develop HCC was at ≥42% non-R^70^-containing sequences and was determined by receiver operating characteristic (ROC) analysis. Using this restriction, high-risk quasispecies (those with ≥42% non-R^70^ codons) were present at baseline in 88.5% (23/26) of the HCC group and in 68.8% (44/64) of the non-HCC group, *P* = 0.06 (Tables 1 and 2 and Fig. 2a).

### Quasispecies at position 91 and HCC risk

A parallel analysis was carried out on position 91 and it was found to be predominantly non-L^91^ in our baseline samples (Figs 1b and 2b), even though L^91^ is the wild type sequence. ROC analysis identified 98.5% non-L^91^ as the cut-point with the greatest ability to classify high-risk and low-risk HCC incidence. Using this cut-point, non-L^91^ quasispecies were present at baseline in 30.8% (8/26) of HCC patients and in 54.7% (35/64) of non-HCC patients (*P* = 0.06) (Tables 1 and 2 and Fig. 2b). Thus, in this series and in contrast to what is observed in Japanese studies^8^,^9^, non-L^91^ quasispecies was determined as a lower risk for HCC incidence in this United States population.

### Cumulative incidence of HCC based on quasispecies at position 70 and 91

**Figure 3:**
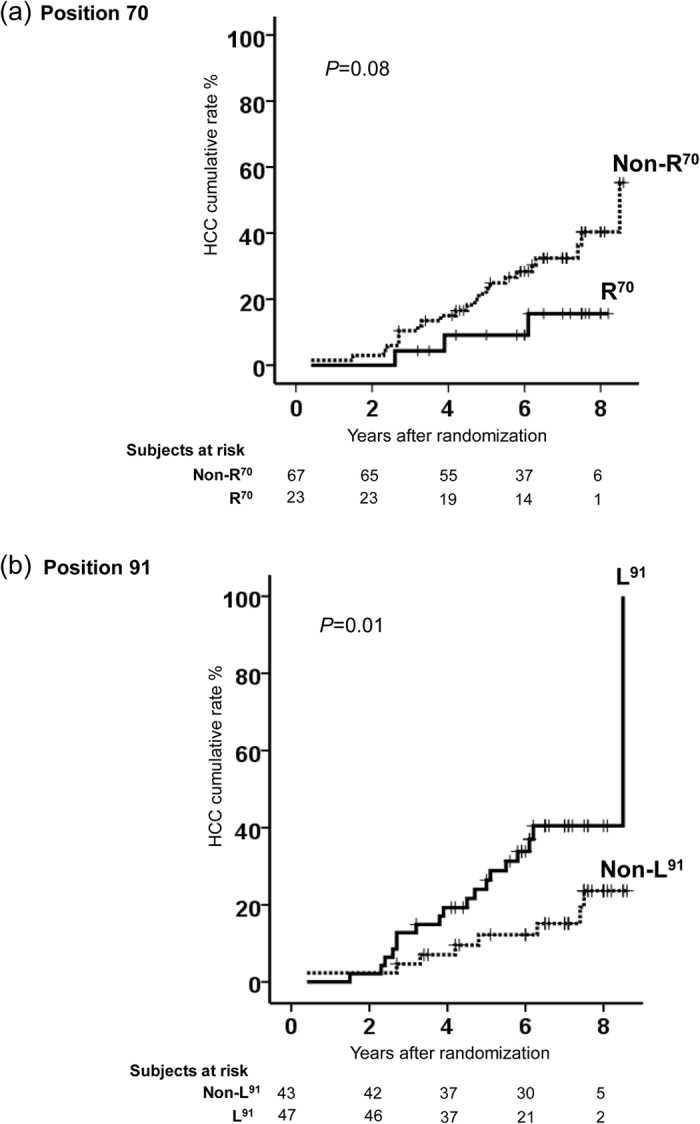
Kaplan-Meier analysis of the cumulative HCC incidence rate based on the frequency of quasispecies at postions 70 (**a**) and 91 (**b**) of the core protein. Cut-points at ≥42% for core-non-R^70^ and at ≥98.5% for core-non-L^91^ were used in this analysis.

To investigate the cumulative incidence of HCC based on point mutations at positions 70 and 91 of the core protein, Kaplan-Meier analysis was carried out. This analysis revealed that the cumulative incidence of HCC for patients infected with quasispecies containing ≥42% non-R^70^ codons versus those infected with quasispecies containing <42% non-R^70^ were 15% and 9%, respectively, at four years; 28% and 9%, respectively, at six years; 40% and 16%, respectively, at eight years (log rank *P* = 0.08) (Fig. 3a). On the other hand, the cumulative incidence of HCC for patients infected with quasispecies containing ≥98.5% non-L^91^versus those infected with quasispecies containing <98.5% non-L^91^ were 7% and 20%, respectively, at four years; 12% and 34%, respectively, at six years; and 24% and 40%, respectively, at eight years (log rank *P* = 0.01) (Fig. 3b).

We also investigated the association between core “haplotypes” (pairs of amino acids at positions 70 and 91 located on the same viral RNA) with HCC development. The distribution of core “haplotypes” (R^70^/L^91^, non-R^70^/L^91^, R^70^/non-L^91^ and non-R^70^/non-L^91^) in patients who developed HCC or not (non-HCC) is shown in Supplementary Fig. 2. None of the haplotypes were significantly associated with HCC using ROC and Fisher’s exact probability test (data not shown).

### Identification of independent factors correlated with HCC risk by Cox regression and matched conditional logistic regression analyses

In order to identify other factors associated with HCC risk, baseline clinical values were analyzed by univariable Cox regression analysis. This identified nine factors with *P* ≤ 0.1: non-R^70^ (≥42% of the quasispecies, *P* = 0.09), non-L^91^ (≥98.5% of the quasispecies, *P* = 0.02), white blood cell count (*P* = 0.004), hemoglobin level (*P* = 0.09), platelet count (*P* = 0.001), AST/ALT ratio (*P* = 0.001), alkaline phosphatase level (<0.001), albumin level (*P* < 0.001) and AFP level (*P* = 0.004) (Table 3). The nine factors were entered in a stepwise backward multivariable Cox regression analysis, which identified one viral factor, non-R^70^ (*P* = 0.04), and three host factors, white blood cell count (*P* = 0.01), AST/ALT ratio (*P* = 0.02) and alkaline phosphatase level (*P* < 0.001), as independently associated with higher HCC risk (Table 3). Additionally, since the study included matched cases and controls, we also performed univariable and multivariable matched conditional logistic regression analyses. Univariable analysis yielded six factors with a *P* ≤ 0.1: white blood cell count (*P* = 0.007), platelet count (*P* = 0.004), the AST/ALT ratio (*P* = 0.01), alkaline phosphatase level (*P* = 0.007), albumin level (*P* = 0.01) and AFP level (*P* = 0.035) (Table 4). Subsequently, these factors were entered into matched conditional logistic regression analysis. This analysis identified only alkaline phosphatase level (*P* = 0.02) as an independent factor associated with a higher HCC risk (Table 4).

## Discussion

In this study, we investigated the association between HCC risk and the percentage of non-R^70^ codons in the viral quasispecies. ROC analysis indicated a cutoff of ≥42% of non-R^70^ as the optimal threshold for correlation with increased HCC risk. Recently, using a modified real-time PCR assay called Q-Invader, Akuta *et al.*, identified a cut-point of ≥20% of non-R^70^ as the optimal threshold for identifying high-risk quasispecies in Japanese patients who later achieved an SVR^17^. The difference between the cut-points in the two studies may be attributed to differences in study design and patient cohorts. The Japanese study evaluated post-SVR HCC risk, while our study evaluated HCC risk among patients who failed antiviral therapy and remained chronically infected. Furthermore, in the Japanese study, the cut-point was determined from samples obtained prior to antiviral treatment. The cut-point in our study was determined on samples collected from patients who had recently failed interferon-based therapy. It has been reported by Kurbanov *et al.* that there is positive selection for non-R^70^ variants during IFN/RBV treatment^27^. In accordance with this, non-R^70^ codons were abundant in our baseline samples. The quasispecies in the majority of the patients in our cohort (77.8%) already had ≥20% non-R^70^ sequences at baseline. Although a significant correlation between non-R^70^ and the unfavorable allele of IL28b have been reported^29^,^30^, we could not investigate this due to the lack of patient consent for IL28b genotyping.

While the HALT-C trial showed that long-term treatment of low dose IFN had no significant impact on disease progression and HCC development in Americans with chronic hepatitis^21^, in contrast, a Japanese study on 494 patients by Takeyasu *et al.* showed that long-term IFN monotherapy significantly reduced the risk of HCC, even in cirrhotic patients^13^. Despite this discrepancy, non-R^70^ of the core protein was associated with increased HCC risk in both studies. In our study, while non-R^70^ was associated with HCC in a Cox regression model, neither non-R^70^ nor non-L^91^ was associated with HCC in a matched conditional logistic regression model. Our study was designed to have three controls for each case, but sequencing revealed that fewer patients than expected had genotype-1b HCV infection and as a result, not all the cases had three controls, reducing the power of the matched analysis.

While the close association between mutations at position 70 with increased risk of HCC and IFN treatment failure is consistent across many epidemiological studies, results are inconsistent regarding the significance of mutations at position 91. In this study, ROC analysis indicated a cut-point of ≥98.5% of non-L^91^ as the optimal threshold for identifying *low* risk quasispecies; there was a tendency toward higher HCC risk among patients in whom the quasispecies contained <98.5% non-L^91^. In contrast, in a Japanese study, non-L^91^ was associated with *higher* HCC risk^8^. Differences in the nature of HCV variants circulating in different geographical regions or with the ethnicity of patient cohorts might account for the disparate findings. In a previous meta-analysis using a multivariable logistic regression model that controlled for Japanese origin, we found that L^91^ was independently associated with increased HCC risk^12^. Future studies are needed to further clarify this issue.

Despite the cumulative epidemiological evidence supporting the impact of core protein mutations on HCV disease progression, particularly HCC, the underlying molecular mechanism is still unclear. To enable mechanistic studies we recently developed a cell culture system for analyzing infectious HCV variants with and without the *core* gene cancer-associated mutations^31^. This system is based on Huh-7 cells cultured long-term in media containing adult human serum rather than fetal bovine serum. Through this straightforward and robust experimental system, we present the first direct evidence for the sequence-specific effects of HCV variants that were first reported in clinical and epidemiological studies. This system will enable further biochemical investigation of the cellular pathways disrupted by high-risk HCV strains. Additionally, we have identified a novel family of HCV core isotypes, referred to as minicores, which contain the C-terminal portion of the classical core protein, but lack the N-terminal portion^32^. Interestingly, the N-termini of two major minicore proteins are at or near positions 70 and 91 and mutations at these positions may alter the function of these minicore proteins and have an impact on HCV pathogenesis.

In conclusion, the results of this study are the first to point out the link between HCV-1b mutations at positions 70 and 91 of the core protein and clinical outcomes in USA patients, justifying the clinical assessment of HCV mutations as prognostic indicators of HCV-induced HCC.

## Additional Information

**How to cite this article**: El-Shamy, A. *et al.* Impact of HCV *Core* gene quasispecies on hepatocellular carcinoma risk among HALT-C Trial patients. *Sci. Rep.* **6**, 27025; doi: 10.1038/srep27025 (2016).

## Supplementary Material

Supplementary Information

Supplementary Information

## Figures and Tables

**Table 1:** Demographic characteristics of HCC and non-HCC patients with HCV-1b infection at baseline.

Factor	HCC (n = 26)	Non-HCC (n = 64)	*P* value
Age	53.9 ± 6.8*	52.6 ± 7.5	0.45^†^
Sex (Female)	10 (38%)	26 (42%)	0.85^‡^
White blood cells (×10^3^/μL)	4.5 ± 1.2	5.6 ± 1.9	0.008
Hemoglobin (g/dL)	14.3 ± 1.5	14.7 ± 1.4	0.27
Platelets (×10^3^/mm^3^)	117 ± 44.0	164 ± 71.3	0.003
AST/ALT ratio	1.07 ± 0.34	0.87 ± 0.27	0.005
Alkaline phosphatase (IU/L)	137 ± 73.3	94.6 ± 35.3	<0.001
Albumin (g/dL)	3.7 ± 0.3	3.9 ± 0.4	0.005
Alpha fetoprotein (ng/mL)	28.7 ± 28.8	15.9 ± 22.5	0.029
Fibrosis score (Ishak score 5 or 6)	15 (58%)	35 (55%)	0.81
HALT-C trial arms (IFN/no-treatment)	13/13	25/39	0.36
High blood glucose (>139 mg/dL)	6 (23%)	16 (25%)	0.85
HCV-RNA (Log10 IU/mL)	6.3 ± 0.6	6.5 ± 0.4	0.13
*Core mutations*			
**Non-R**^**70**^			
≥42% of scored quasispecies^**††**^	23 (88.5%)	44 (68.8%)	0.06
**Non-L**^**91**^			
≥98.5% of scored quasispecies^**††**^	8 (30.8%)	35 (54.7%)	0.06

^*^Mean ± S.D, ^†^ *t* test, ^‡^ Fisher’s exact test, ^††^ optimal cut-point of quasispecies proportion determined by ROC analysis.

**Table 2:** Relationship between HCC incidence and various cut-off points of percent non-R^70^ and non-R^91^ quasispecies.

Core mutations	Quasispecies cut-off point	HCC (n = 26)	Non-HCC (n = 64)	*P* value‡
Non-R^70^	≥10% of scored quasispecies	24 (92.3%)	48 (75%)	0.08
	≥20% of scored quasispecies	23 (88.5%)	47 (73.4%)	0.16
	≥40% of scored quasispecies	23 (88.5%)	45 (70.3)	0.1
	≥42% of scored quasispecies^**††**^	23 (88.5%)	44 (68.8%)	0.06
	≥50% of scored quasispecies	21 (80.0%)	43 (67.2%)	0.3
	≥90% of scored quasispecies	20 (76.9%)	39 (60.9%)	0.2
Non-L^91^	≥10% of scored quasispecies	20 (76.9%)	56 (87.5%)	0.2
	≥20% of scored quasispecies	20 (76.9%)	56 (87.5%)	0.2
	≥50% of scored quasispecies	19 (73.1%)	56 (87.5%)	0.12
	≥90% of scored quasispecies	19 (73.1%)	55 (85.9%)	0.2
	≥98.5% of scored quasispecies^**††**^	8 (30.8%)	35 (54.7%)	0.06

^‡^Fisher’s exact test, ^††^Optimal cut-point of quasispecies proportion determined by ROC analysis.

**Table 3:** Cox regression model to identify independent risk factors correlated with HCC risk among HALT-C patients with HCV-1b infection.

Variable	Univariable		Multivariable	
	Hazard ratio (95% CI)	*P* value	Hazard ratio (95% CI)	*P* value
Non-R^70^ (≥42% of scored quasispecies)	2.76 (0.83–9.23)	**0.09**	3.83 (1.02–14.3)	**0.04**
Non-L^91^ (≥98.5% of scored quasispecies)	0.36 (0.15–0.84)	**0.02**		
Age (per year)	1.02 (0.97–1.08)	0.42		
Sex (Female)	0.94 (0.43–2.08)	0.89		
White blood cells (per 10^3 ^cell/μL)	0.66 (0.5–0.88)	**0.004**	0.64 (0.45–0.89)	**0.01**
Hemoglobin	0.78 (0.59–1.04)	**0.09**		
Platelets (per 10^3 ^cells/mm^3^)	0.98 (0.97–0.99)	**0.001**		
AST/ALT ratio	5.56 (2.01–15.37)	**0.001**	4.08 (1.24–13.47)	**0.02**
Alkaline phosphatase (per IU/L)	1.01 (1.0–1.02)	**<0.001**	1.01 (1.0–1.02)	**<0.001**
Albumin (per g/dL)	0.16 (0.06–0.44)	**<0.001**		
Alpha fetoprotein (per ng/mL)	1.02 (1.0–1.04)	**0.004**		
Fibrosis score (Ishak score 5 or 6)	1.22 (0.56–2.66)	0.62		
High blood glucose >139 mg/dL	0.9 (0.35–2.19)	0.78		
HCV-RNA (per Log10 IU/mL)	0.54 (0.23–1.26)	0.16		

**Table 4:** Matched conditional logistic regression model to identify independent risk factors correlated with HCC development among HALT-C patients with HCV-1b infection.

Variable	Univariable		Multivariable	
	Hazard ratio (95% CI)	*P* value	Hazard ratio (95% CI)	*P* value
Non-R^70^ (≥42% of scored quasispecies)	2.22 (0.66–7.5)	0.2		
Non-L^91^ (≥98.5% of scored quasispecies)	0.69 (0.25–1.98)	0.49		
Age (per year)	1.03 (0.96–1.1)	0.4		
Sex (Female)	1.04 (0.4–2.69)	0.94		
White blood cells (per 10^3 ^cell/μL)	0.59 (0.41–0.87)	**0.007**		
Hemoglobin (per g/dL)	0.81 (0.58–1.11)	0.19		
Platelets (per 10^3 ^cells/mm^3^)	0.98 (0.97–0.99)	**0.004**		
AST/ALT ratio	11.9 (1.81–78.5)	**0.01**		
Alkaline phosphatase (per IU/L)	1.02 (1.01–1.04)	**0.007**	1.02 (1.0–1.04)	**0.02**
Albumin (per g/dL)	0.14 (0.03–0.64)	**0.01**		
Alpha fetoprotein (per ng/mL)	1.02 (1.0–1.05)	**0.035**		
High blood glucose >139 mg/dL	1.05 (0.37–2.96)	0.93		
HCV-RNA (per Log10 IU/mL)	0.44 (0.15–1.34)	0.15		
