# Supplementary material for: Impact of HCV core gene quasispecies on hepatocellular carcinoma risk among HALT-C trial patients
Source: Sci Rep. 2016 Jun 1;6:27025. doi: 10.1038/srep27025 (PMC4887904; doi:10.1038/srep27025)
Supplement: Supplementary Information [file srep27025-s2.pdf]

THE HALT-C TRIAL

Extension of the **H**epatitis C **A**ntiviral **L**ong-term **T**reatment against **C**irrhosis (HALT-C) Trial: A randomized controlled trial to evaluate the safety and efficacy of long-term peginterferon alfa-2a for treatment of chronic hepatitis C in patients who failed to respond to previous interferon therapy.

Study Sponsor: NIDDK

Project Officer: James Everhart, M.D., M.P.H.

Co-Project Officer: Patricia R. Robuck, Ph.D., M.P.H.

Scientific Advisor: Leonard Seeff, M.D.

Version Date: July 21, 2006

Principal Investigators:

HCV Treatment Centers:
Gyongyi Szabo, M.D.
University of Massachusetts
Worcester, MA

Satellite Site of UMASS
Herbert Bonkovsky, M.D.
University of Connecticut
Farmington, Connecticut

Adrian Di Bisceglie, M.D.
Saint Louis University
Saint Louis, MO

Jules Dienstag, M.D.
Massachusetts General Hospital
Boston, MA

Gregory T. Everson, M.D.
University of Colorado Health Sciences Center
Denver, CO

Marc Ghany, M.D.
NIDDK/LDS
Bethesda, MD

Timothy Morgan, M.D.
University of California -Irvine
Orange, CA

William M. Lee, M.D.
University of Texas Southwestern
Dallas, TX

Karen L. Lindsay, M.D.
University of Southern California
Los Angeles, CA

Anna Lok, M.D.
University of Michigan
Ann Arbor, MI

Mitchell L. Shiffman, M.D.
Virginia Commonwealth University
Richmond, VA

Virology Core Laboratory:
David Gretch, M.D., Ph.D.
University of Washington
Seattle, WA

Data Coordinating Center:
Kristin K. Snow, Sc.D.
New England Research Institutes
Watertown, MA

TABLE OF CONTENTS

PROTOCOL....4
A. PURPOSE AND OVERVIEW....4
A.1. Purpose:....4
A.2. Overview:....4
B. BACKGROUND....5
B.1. Natural history of chronic hepatitis C:....5
B.2. Response to interferon or interferon-ribavirin therapy:....8
B.3. Histologic improvement during treatment with interferon:....9
B.4. Effect of interferon therapy on development of hepatocellular carcinoma:....9
B.5. Pegylated interferon (peginterferon):....10
B.6. Summary and Study Rationale:....11
C. HYPOTHESES:....12
D. SPECIFIC AIMS:....12
E. INCLUSION CRITERIA....13
F. EXCLUSION CRITERIA....15
G. SCREENING OF PATIENTS....18
G.1. Identification of potential patients....18
G.2. Screening of patients....19
G.3. Screening visit #1:....19
G.4. Screening visit #2:....20
H. LEAD-IN PHASE:....21
H.1. Baseline visit:....21
H.2. Weeks 2-20....22
I. CONTINUED FOLLOW-UP OF WEEK 20 RESPONDERS....22
I.1. Eligibility for continued treatment at week 24:....22
I.2. Treatment discontinuation:....22
I.3. Patient Visits:....22
I.4. Management:....22
J. RANDOMIZED PHASE....23
J.1. Assessment for Randomization....23
J.2. Follow-up schedule:....24
J.3. Liver biopsy:....24
J.4. Post-treatment follow-up:....24
J.5. Endoscopy:....25
K. OUTCOME VARIABLES....25
K.1. Primary Outcome Variables....25
K.2. Secondary Outcome Variables....25
L. CESSATION OF TREATMENT....25
L.1. Outcomes which require permanent discontinuation of treatment:....25
M. DOSE MODIFICATION:....26
M.1. Peginterferon alfa-2a dosing:....26
M.2. Ribavirin dosing:....27
M.3. Pregnancy:....28
N. ADVERSE EVENTS....28
N.1. Definition....28
N.2. Data collection procedures for adverse events....29

N.3. Reporting procedures....29
O. SAMPLE SIZE....30
O.1. Estimates of rates for the primary outcome variable....30
O.2. Sample size estimate....30
O.3. Power Analyses....31
P. DATA MANAGEMENT AND ANALYSIS....31
P.1. Data Management....31
P.2. Analysis of Baseline Data....32
P.3. Analyses of Lead-in Phase....32
P.4. Analyses of the Primary Outcome....32
P.5. Interim analyses....33
P.6. Analyses of Secondary Endpoints....34
Q. POLICIES AND PROCEDURES....34
Q.1. Trial Organization....34
Q.2. Changes to the Protocol....37
Q.3. Publication Policies....37
Q.4. Ancillary Studies:....37
TABLES AND FIGURES....38
Figure 1: Design of the HALT-C Trial....38
Figure 2: Trial Organization....38
Figure 2: Trial Organization....39
Table 1: Trial Flow Chart – Screening & Lead-in Phase (Lead-in Group)....40
Table 2: Trial Flow Chart – Randomized Phase....41
Table 3: Trial Flow Chart – Week 20 Responders....413
Table 4: Trial Flow Chart – Screening & Randomization Visit (Express Group)....43
REFERENCES....44
APPENDICES....53
A. Definitions....53
B. Child-Turcotte-Pugh Score for Grading Severity of Liver Disease....55
C. Management Guidelines....56
C.1. Depression....56
Attachment 1....60
C.2. HCC Screening....61
C.3. Portal Hypertension....62
Attachment 2....65
D. Adverse Events....66
D1. Relationship of adverse event to trial medication....66
D2. Adverse event pattern of events....67
D3. Adverse event status....67
D4. Peginterferon alfa-2a Dose Adjustment Guidelines....67
E. Screening Consent Form....72
F. Trial Consent Form....72

PROTOCOL

A. PURPOSE AND OVERVIEW

A.1. Purpose:

The purpose of this study is to conduct a randomized, controlled trial to determine if long-term interferon therapy can reasonably reduce the risk of histologic progression to cirrhosis, decompensated liver disease and/or hepatocellular carcinoma in patients with chronic hepatitis C and advanced fibrosis or cirrhosis who failed to respond to previous interferon therapy.

A.2. Overview:

Up to 1800 patients who meet the inclusion and exclusion criteria (Sections E and F) will be entered into a Lead-in Phase. They will be treated with a combination of peginterferon alfa-2a and ribavirin for a period of 24 weeks. Patients who have no detectable HCV RNA at week 20 will continue on combination treatment until week 48. Patients who do not clear virus will be randomized 50:50 at week 24 to receive either peginterferon alfa-2a alone or no further therapy for the next three and a half years. Nine hundred patients will be randomized and both randomized groups will be monitored quarterly during these 42 months. Biopsies for these patients will be obtained at 24 and 48 months after the start of the Lead-in Phase.

In August, 2001, to increase enrollment two modifications were made to the study design: Those Week 20 Responder patients with virologic breakthrough or relapse documented by detection of HCV RNA at weeks 36, 48, 60 or 72, and those patients who are adequately treated with pegylated interferon and ribavirin combination therapy outside of the HALT-C Lead-in (Express Group) will be considered for randomization into the maintenance phase. These patients will be followed for an additional 48 months and their biopsies performed 18 months and 42 months after randomization.

All treatment in the randomized phase will end in January 2007. A small number of patients may not have completed 48 months at that time. Every patient will stop treatment at that time point, regardless of what month of randomization they are in. Those patients who complete Month 48 will be offered an "extended follow-up" visit after their Month 54 study visit at Month 60 and Month 72 through April 2007. These visits will primarily be to identify outcome events, and to provide information to patients concerning the current status of the trial. Some questionnaires, blood tests, and an ultrasonogram will be performed. This visit is not to take the place of patients establishing a relationship with a primary care or liver specialist physician once the Month 54 visit is completed.

Those patients who are currently being followed will be offered visits at 6 month intervals through October 2009. Depending on when the patient was randomized this visit will be a Month 54, Month 60, Month 66, Month 72, Month 78, Month 84, Month 90, Month 96, Month 102 and Month 108. Each of these visits will be similar. These visits will primarily be to identify outcome events, and to provide information to patients concerning the current status of the trial. Some questionnaires, blood tests, and an

ultrasonogram will be performed. This visit is not to take the place of patients establishing a relationship with a primary care or liver specialist after April 2007 visit is completed.

B. BACKGROUND

B.1. Natural history of chronic hepatitis C:

Chronic hepatitis C, an illness caused by the hepatitis C virus (HCV), affects 4 million patients in the USA, and results in 10,000 deaths annually (1,2). Hepatitis C is the most common cause of liver transplant (3, 4) and is a major predisposing factor to the development of hepatocellular carcinoma (HCC) (3,4-9) in the United States. Additionally, hepatitis C produces debilitating fatigue in 12% of patients and a variety of extra-hepatic manifestations in 1% (chronic renal failure, hypothyroidism, etc.) (10). Recent evidence is consistent with a large population of undocumented hepatitis C patients that will yield triple the number of cases annually of hepatocellular carcinoma and decompensated liver disease in the next 20 years (1).
The natural history of chronic HCV infection has not yet been fully defined (11). Current data suggest that the process runs an indolent course during the first two decades after initial infection, accounting for little morbidity and mortality. Serious sequelae are more likely to emerge as the disease process enters the third and fourth decades after infection. Three approaches have been used: the prospective study, the retrospective study, and the combined retrospective-prospective, otherwise referred to as non-concurrent prospective study. The prospective study would constitute the ideal approach but, as already noted, it suffers from the fact that the onset of the acute illness is rarely recognized and that the follow-up period needed is markedly protracted, lasting three or more decades, a difficult commitment for an individual investigator.

Currently, much of the reported data on the natural history of hepatitis C accrue from retrospective studies of persons with already established chronic liver disease, excluding from analysis the group that does not reach clinical awareness (12). Five prospective studies of transfusion-associated non-A, non-B hepatitis have been reported from the United States and Europe (9,13-16), the duration of evaluation ranging between 8 and 14 years. Clinical symptoms of varying type and severity were identified in 4% to 13% of patients, liver biopsies revealed the presence of cirrhosis in 16% to 24%, Hepatocellular carcinoma was reported in two of the studies (0.7% and 1.3%, respectively), and death attributed to liver disease was reported in between 1.6% and 6.0% over this period, averaging about 3%.

Although none of these five studies included a non-infected control group, and periods of follow-up were somewhat brief for the usually protracted course of chronic hepatitis C, the studies yielded important information. They demonstrated that over periods of about a decade to a decade and a half from acute onset, morbidity and mortality from liver disease could be clearly demonstrated, although at a relatively modest frequency.

Clinical Events of Chronic HCV

The majority of the clinical consequences of chronic liver disease (CLD) are related to the effect of progressive hepatic fibrosis in: 1) producing portal hypertension and 2) progressive decline in the functioning hepatic mass. Portal hypertension is usually present by the early cirrhotic stage and the amount of functioning hepatic tissue (functional mass) progressively decreases as the disease progresses from early to advanced cirrhosis (17,18). The interaction of these two factors produces most of the clinical outcomes of chronic liver disease. Thus, hypersplenism (19), marked collateral formation (with esophageal varices) and hepatic congestion related to portal hypertension characterize the cirrhotic patient (12-15,20-23). Progressive CLD finally results in the major clinical events of variceal bleeding and ascites formation (12,15,20,21). A minimum portal pressure gradient ≥12 mm/Hg is required to initiate these clinical events, but the average portal pressure gradient at onset of these problems is greater than this (17 ± 3 mm/Hg). (24-26). Although most cirrotic patients have varices, fewer than 30% of patients have variceal bleeding. The size and appearance of varices correlates with the tendency for bleeding (27). A patient's tolerance of a bleeding episode is related to the hepatic reserve as assessed by Child's class.

Ascites is a major clinical process that occurs spontaneously at the moderately advanced cirrhotic stage at a point when the functional mass is approximately half of normal (28-30). The 5 year survival after the onset of ascites is 20% (31-33). Further progression of liver disease leads to refractory ascites, hepatic encephalopathy, spontaneous bacterial peritonitis and renal dysfunction (34-38, 39). The one-year survival is less than 50% in association with these clinical problems.

HCC is the cause of death in 10-20% of cirrhotics with chronic HCV and nearly all of these patients are at the cirrhotic stage (4-8). Iron overload, co-infection with other viruses, co-existent liver diseases and genetic factors may increase the likelihood of HCC (4-6,8). Symptomatic tumors are generally large and lead to an average survival of 3-4 months. There is no effective therapy for large tumors. Screening for HCC with alpha-fetoprotein (AFP) and ultrasound increases the detection of small tumors (40) that may be treated with surgery or alcohol injection (41,42).

Prognostic Models

The natural history and the prognostic factors in hepatic cirrhosis have been extensively studied but several aspects remain unclear and prognostic factors have not been validated fully (20,32,33,43-58). The level of hepatic fibrosis, Child-Turcotte-Pugh score, blood tests and/or the presence of clinical problems are traditional ways to assess prognosis. Most histologic scoring systems have cirrhosis as a single stage and the mean 10 year survival in patients with compensated cirrhosis is 50% (31,32,59). Clinical problems (particularly ascites) indicate patients with a poor prognosis. In the studies discussed above, those patients with compensated cirrhosis who developed ascites had a 2 year survival of 50% and 5 year survival of 20% (31-33). Various scores and models combining biochemical and histologic data have been proposed (45,46). One of these, the Child-Turcotte-Pugh score, has been shown to be a simple and valid index for assessing the severity of disease and prognosis in patients with cirrhosis (47-49).

Measurement of the functional hepatic mass could be useful in determining prognosis. Quantitative tests of liver function, such as the aminopyrine breath test, indocyanine clearance, as well as radiographic studies such as MRI, ultrasound, and CT scan have been evaluated in detection of cirrhosis and prognosis (30,60-65). Studies have demonstrated that the perfused hepatic mass (PHM) by quantitative liver spleen scan faithfully provides information regarding reserve hepatic function in patients with chronic liver disease (17,18,29,66). Quantitative expression of sulfur colloid distribution between liver, spleen and bone marrow expressed as PHM correlates with the severity of chronic liver disease by histologic assessment (17,29), severity of cirrhosis at peritonoscopy (66), and the functional hepatic mass of the explanted liver at transplant (66). The rate of decline in these functional indices may provide important insight into the natural history of HCV.

Factors Affecting Natural History

The major determinant of progressive liver disease in patients with chronic hepatitis C is hepatic inflammation which stimulates the progressive buildup of fibrosis (9,10,12-14,20-22,45,67-69). This process generally takes 20-40 years from initiation of infection to death from cirrhosis and can be markedly accelerated by alcohol (3,13-15,22,23). Other factors that are associated with a more rapid rate of progression are a high grade of inflammation, longer duration of disease and age (20). Male sex, iron overload and hepatic fat may be other risk factors for more rapid progression of fibrosis. The rate of fibrosis has been estimated in a selected population of patients with hepatitis C to be .133 METAVIR fibrosis units per year (20,70).

Viral factors that might determine outcome include the dose at the time of infection, genotype, and the presence of quasi-species (HCV genetic diversity). The effect of dose in initiating progressive disease is unknown (71,72). Most studies have shown that genotype 1 provokes a more severe disease than does genotype 2 (73,74) and that 1b is more harmful than 1a. (53). The impact of HCV quasi-species was studied by comparing its presence among those with acute resolving hepatitis with those who progress to chronic hepatitis (75,76).

Host-related factors that might play a role include age at the time of infection, race, and gender. Genetic predilection warrants further study. With respect to age, the rate of disease progression seems to increase the older the age at the time of infection (20).

Extraneous factors that may conceivably promote progression include super-infection with other viruses (77-82), smoking (83), exposure to defined or undefined environmental contaminants, or concomitant or pre-existing chronic alcoholism. Studies using univariate and multivariate regression analyses have suggested that chronic hepatitis C is worsened by infection with hepatitis B virus (84); this increases the risk of carcinogenesis as well (83,85).

An important co-factor in progression of chronic hepatitis C to cirrhosis and HCC is that of chronic alcoholism, even if it had existed only in the past. The relationship between HCV infection and alcoholic liver disease was first noted in early

epidemiological surveys (86,87). It is now apparent that alcoholism may promote HCV replication (88,89). Numerous surveys using regression analysis target alcohol as a major factor in promoting progression of liver disease in persons with chronic HCV infection (90-94).

B.2. Response to interferon or interferon-ribavirin therapy:

Virologic cure has been the major goal of treatment strategies. However, decrease in progression of fibrosis and attenuation of serious clinical events are secondary goals. Evidence supports the value of interferon in both aspects.

Virologic Cure
Interferon is the only medication acting either alone or in combination with other drugs that can cure a patient of chronic hepatitis C (95-97) through both anti-viral and immunologic mechanisms (98,99). The four types of interferons that have been evaluated in a large number of patients with chronic HCV infection have comparable safety and efficacy in the treatment of chronic HCV infection (97,100-103). However, less than half of the patients with chronic hepatitis C treated with interferon-alfa monotherapy develop a negative HCV RNA during treatment. Nearly all patients who respond to interferon do so within the first 3-4 months (104). No more than 20% have a sustained benefit from the initial course of interferon-alfa treatment as indicated by persistently normal SGOT/SGPT and approximately 11% of these will have virologic cure (105).

More potent anti-hepatitis C therapies have been developed. Combination therapy with interferon and ribavirin is more effective than interferon alone in inducing virologic, biochemical, and histologic improvement in both naïve and relapsed patients with chronic hepatitis C (106-111). A sustained virological response of 38% and a sustained biochemical response of 36% has been demonstrated when this combination was used in naive patients (106). The response rate was better for non-1 genotype than genotype 1 (66% vs 28%) (106) and for low viral titer, (<2M) as opposed to high viral titers (43% vs 36%) (106). Combination therapy is well tolerated: side effects are similar to interferon alone, with the additional risk of hemolytic anemia (111). Twenty-four weeks of treatment with Intron-A plus ribavirin in patients who relapse following initial therapy with Intron-A yield a 48% sustained response. Similarly, consensus interferon (CIFN) in a dose of 15 μg TIW for 48 weeks demonstrated a 13% sustained response rate in previous non-responders and a 58% response rate in previous relapsers (100,112,113,114-116). However, despite improved therapy, more than 50% of all patients treated fail to clear HCV permanently.

Prevention of Progression and Adverse Clinical Outcomes
Although the major goal of therapy has been virologic cure, this is achieved in <40% of patients. However, patients treated with mono-therapy over the last 12 years appear to obtain secondary clinical benefits even without sustained virologic response. Patients without a virologic response to interferon still showed a significant histologic improvement in most studies (114,117-127). In patients treated with a course of interferon, the rate of progression from pre-cirrhosis to cirrhosis is decreased (128-138), the incidence of decompensation with ascites is reduced (59,128-138) and HCC

is less frequent (59,139-142). In general, the benefit of therapy is greater in those who have a sustained response, compared to relapsers or non-responders (59). However, even non-responders with cirrhosis have a better survival. Thus, interferon treatment may be anti-fibrotic and prevent or slow the progression of disease. Several studies have demonstrated the effectiveness of a continuous low dose of interferon for patients with chronic hepatitis C (103,143,144), raising the question of whether the benefit of treatment could be better sustained by long-term therapy.

B.3. Histologic improvement during treatment with interferon:

Improvements in liver histology have been observed in numerous studies in which pre- and post-interferon treatment liver biopsies have been performed. Such improvements have not only been observed in patients who achieved a sustained virologic response, but in non-responding patients, who remain viremic, as well. In general, a histologic response to interferon therapy occurs in approximately 80% of patients who have achieved a biochemical and/or virologic response to interferon therapy and in about 40% of interferon non-responders (145-152). Following a relatively brief, 6-12 month course of interferon, these histopathologic changes appeared to be confined to improvements in hepatic inflammation, with little change in hepatic fibrosis. However, since it is believed that hepatic inflammation drives progression to fibrosis (153-157), these observations suggest that long term interferon therapy may slow progression to more extensive fibrosis or cirrhosis.

A recent study has evaluated the hypothesis that long term interferon therapy, in those patients who remain virologic non-responders, may indeed prevent histologic progression (158). In this study patients underwent repeat liver biopsy following an initial 6-month course of interferon therapy. Those patients who exhibited a histologic response were randomly selected to either discontinue treatment and be followed prospectively over an additional two years, or to remain in interferon treatment at a dose of 3 mu TIW. After 2 years of continuous interferon therapy hepatic inflammation remained significantly less than at the pre-treatment baseline and hepatic fibrosis declined. In contrast, patients who discontinued interferon therapy had an increase in hepatic inflammation back to the pre-treatment baseline and an increase in hepatic fibrosis was observed after 2 years follow-up. A larger controlled randomized trial is now needed to confirm these preliminary observations.

B.4. Effect of interferon therapy on development of hepatocellular carcinoma:

A provocative report by Nishiguchi et al (159) suggested that treatment of cirrhotic patients with interferon reduced the incidence of HCC over a mean of 4.4 years from 38% in untreated controls to 4% in treated patients. What was most surprising about this report were the facts that a) such a profound impact over many years resulted from such a limited, six-month course of interferon therapy and b) the beneficial effect on incidence of HCC was not limited to interferon responders but occurred as well in patients who had not achieved biochemical or virologic responses. Schalm et al (160) summarized three studies on the impact of interferon therapy on the occurrence of HCC in cirrhotic patients with hepatitis C (159,161-162). Taken together, these three studies included 272 untreated patients, 371 nonresponders, and 60 sustained responders after interferon treatment, and the frequency of HCC in these three groups

was 15%, 4%, and 0%, respectively, suggesting an apparent beneficial effect of interferon treatment in preventing HCC in cirrhotic patients with hepatitis C. Studies with similar results continue to appear in the literature. (163)

At first glance, these data appear to provide strong support for the hypothesis that interferon therapy in cirrhotic patients with hepatitis C prevents HCC. Multivariate analysis, however, revealed that clinical differences at the time of entry, not interferon therapy, correlated with the incidence of HCC (160). In fact, progression from cirrhosis to HCC may be associated with such factors as genotype 1b (164-165) male gender, and age >60 (164), supporting the hypothesis that host and virus variables may play a more important role in the development of HCC than antiviral therapy. A similar conclusion was reached by Bruno et al (164) in a retrospective analysis of the incidence of HCC among 163 cirrhotic patients with hepatitis C. The apparent reduction of HCC in interferon-treated patients represented a treatment bias towards patients with less advanced disease.

Although the treatment bias in these studies does not support a role for interferon in preventing HCC, they do not prove that treatment will fail to be effective. The hypothesis remains viable that reduction of liver injury by antiviral therapy could slow the progression of fibrosis to cirrhosis, reduce the rate of new fibrosis in cirrhotics, and, as a consequence, reduce the likelihood of decompensation and even delay the onset of HCC. The only way to test this hypothesis adequately is in a prospective controlled trial. Given that a 1-4% annual incidence of HCC has been observed in cirrhotic patients with chronic hepatitis C (164-167), a prospective trial of sufficient study size and duration would have the power to identify an interferon-associated reduction in HCC.

B.5. Pegylated interferon (peginterferon):

A candidate drug to be used for long-term antiviral therapy must have an acceptable safety/tolerability profile. Considerable experience and safety data are available for interferon, and many patients have been treated for many years without an appreciable increase in adverse events over time. Ribavirin is now included routinely in combination therapy for chronic hepatitis C, but its safety profile when taken over several years has not been established. Because ribavirin causes hemolysis in a patient population with cirrhosis, leading to anemia, the risks of using the drug will be increased. Therefore, the use of combination therapy, especially for the four-year duration of this trial, is not planned.

Although standard preparations of interferon are available as candidate treatments for this trial, we propose the use of peginterferon, a long-acting preparation of interferon-alfa conjugated to polyethylene glycol (PEG). Preliminary observations with peginterferon suggest that it is absorbed at rates slower than that of interferon-alfa, with a delayed Tmax and a prolonged concentration peak, achieved primarily as a result of the much longer (median seven-fold longer) elimination time of peginterferon. In addition, drug concentrations remain more stable and sustained over time than they do with more frequent administration of shorter-acting preparations with their resultant peaks and troughs. Trials of peginterferon given by subcutaneous injection once a

week have shown levels of biochemical and virologic response comparable to those achieved with standard interferon-alfa given three times a week. Moreover, a recent trial of peginterferon among previously untreated patients with chronic hepatitis C suggested that the long-acting interferon may be more effective than standard interferon. Among patients treated with peginterferon, the sustained response rate measured six months after completion of therapy was 36% (168), which is comparable to the efficacy reported for combination interferon-ribavirin therapy (169-170). By relying on therapy that can be administered once a week, we expect a higher patient acceptability, and therefore a longer enrollment and better compliance.

Data available indicate that adverse events associated with peginterferon are comparable to those observed in patients treated with standard interferon-alfa, and in multiple-rising-dose studies, fewer patients taking peginterferon reported adverse effects compared to those taking standard interferon-alfa. Recent experience in dose-finding studies suggest an increase in thrombocytopenia and neutropenia among peginterferon-treated patients compared to interferon-alfa-treated patients, although seldom leading to dose modification or discontinuation. Therefore, more vigilant platelet-count and WBC monitoring will be necessary in a trial of peginterferon, especially in cirrhotic patients.

B.6. Summary and Study Rationale:

Chronic, low-grade inflammation and liver injury due to hepatitis C infection leads to fibrosis and the development of cirrhosis in a significant portion of infected patients. Although viral eradication has been the primary goal of treatment of hepatitis C thus far, patients who are non-responders constitute 50% of all those with hepatitis C. The evidence presented here suggests that interferons may slow or arrest progression of injury-related fibrosis, even in non-responder patients who fail to clear the virus. Regardless of whether these potential benefits are related to viral replication or to a direct anti-fibrotic mechanism, this potential benefit of interferon therapy needs further confirmation.

The aim of the present study is to determine whether prolonged interferon-based therapy can be achieved and maintained in a reasonable number of subjects over several years' time, and whether the anticipated benefits are worth the risk and expense involved. The study population will be limited to those at highest risk of progression to cirrhosis or its complications, namely, patients with established fibrosis or cirrhosis on biopsy at study entry. Since all patients enrolled will have had different previous treatments, the present study design provides that all patients entering the long-term portion of the study have begun at a common starting point and have had a second chance at reaching a sustained virologic response. In the initial or lead-in course of therapy all patients will be treated with peginterferon plus ribavirin for a period of 24 weeks, with virologic assessment at 20 weeks to refute their non-responder status. Thus, patients entering the long-term study will know that they have been given the very latest regimen in an attempt to achieve virologic clearance and, having failed, now can be certain that they are non-responders to conventional therapy It is anticipated that approximately 20% will have no detectable virus in serum at the 20 week point, and that this group may continue treatment and receive a full course of

48 weeks' therapy. Those who remain HCV-positive in serum at 20 weeks will be randomized to receive either continued peginterferon (without further ribavirin) or no further treatment beyond careful observation for the ensuing 3 and 1/2 years. Week 20 responders who develop detectable HCV RNA at weeks 36, 48, 60 or 72 may be randomized to receive either continued peginterferon (without further ribavirin) or no further treatment beyond careful observation for an additional 42 months.

Since patients with both fibrosis and cirrhosis will be included, therapeutic endpoints will be of two types: histologic progression of disease as well as development of evidence of cirrhotic decompensation. Ancillary studies will help define the role of interferon in slowing the evolution of portal hypertension and the role of fat change in disease progression. The extent of side effects of interferon as well as a detailed assessment of quality of life will be obtained for all study participants. Further studies will be aimed at increasing our understanding of the complex interactions of virus and host in this intriguing disease.

The purpose of the continued observation in this long-term follow-up phase of the HALT-C Trial is to determine the outcome of patients enrolled in this study over a longer time interval.

C. HYPOTHESES:

C.1. In patients with chronic hepatitis C who failed to respond to previous interferon therapy, long term treatment with interferon can safely prevent progression of advanced fibrosis to cirrhosis.

C.2. In patients with cirrhosis secondary to chronic hepatitis C who failed to respond to previous interferon therapy, long term treatment with interferon can safely:

C.2.a. Reduce the risk of hepatic decompensation.

C.2.b. Reduce the risk of developing hepatocellular carcinoma.

D. SPECIFIC AIMS:

D.1. To determine if 4 years of interferon therapy will prevent progression of advanced fibrosis to cirrhosis in patients with chronic hepatitis C who failed previous interferon treatment.

D.2. To determine if 4 years of interferon therapy, in patients with cirrhosis secondary to chronic hepatitis C who failed previous interferon treatment, will:

D.2.a Reduce the risk of developing hepatic decompensation;

D.2.b Reduce the need for hepatic transplantation;

D.2.c Reduce the risk of developing hepatocellular carcinoma; and

D.3. To determine if 4 years of interferon therapy will improve the quality of life in patients with advanced fibrosis or cirrhosis secondary to chronic hepatitis C who failed previous interferon treatment.

E. INCLUSION CRITERIA

Patients can be screened for the HALT-C Trial as either “Lead-in” or “Express” patients. Lead-in patients are treated for 24 weeks with peginterferon alfa-2a and ribavirin as part of the HALT-C Trial. Patients who have been treated with peginterferon and ribavirin outside of the HALT-C Trial can bypass the Lead-in Phase and be randomized as “Express” patients.

Sections E1, E2, E5, E8, and E9 are for all patients. Sections E3, E4, and E6 are for “Lead-in” patients and E7 is for “Express” patients.

E.1. Age at entry at least 18 years.

E.2. HCV-RNA positive or serology positive for HCV antibody by a second generation or higher assay.

E.3. Lead-in patients must have had previous treatment with any interferon preparation (standard or pegylated interferon), utilized either alone or in combination with ribavirin, administered at a minimum dose of 3 mU three times weekly or its equivalent, for at least 12 weeks. No interferon, ribavirin, or amantadine treatment in the 2 months prior to screen visit #1.

E.4. Lead-in patients: Documented non-response to the most recent adequate course of interferon therapy, as defined in E.3. Non-response will be defined as follows:

E.4.a. A positive test for HCV RNA after at least treatment week 12 of adequate interferon therapy while still being treated, or within one week of discontinuing the most recent adequate course of interferon.

E.4.b. If virologic measurements were not performed or are unavailable from the most recent adequate treatment, serum ALT must have been elevated within 6 months of onset and at anytime during 4 weeks prior to or 1 week after stopping this prior adequate course of interferon therapy.

E.4.c. If elevated serum ALT’s or positive HCV-RNA laboratory test are not available, a clinical note documenting non-response will suffice.

E.5. A positive test for HCV RNA, by the Core Virology Laboratory, at the time of screening to enter the study.

E.6. Lead-in patients must satisfy the following biopsy criteria:

E.6.a. Patients selected to enter the HALT-C Lead-In Phase, must have had a liver biopsy performed at least 2 months following the last course of interferon and within 12 months prior to the baseline visit, demonstrating at least Ishak stage 3 fibrosis as judged by the clinical center pathologist.

E. 6. b. Patients with a history of Ishak stage 3 or higher fibrosis, who subsequently have fibrosis scored as Ishak 2 can also be enrolled. Both biopsies must be read by the local HALT-C pathologist using the Ishak fibrosis scoring system. The most recent Ishak fibrosis score will be used for determination of the study outcome.

E.7. Express patients: Documented adequate pegylated interferon and ribavirin for at least 24 weeks may be substituted for the Lead-in Phase of the study provided the following criteria are met:

E.7.a. adequate drug dosing is defined as follows:

1. intent to treat with
   a. peginterferon alfa-2a 180 mcg/wk or peginterferon alfa-2b 1.5 mcg/kg/wk

   AND

   b. 800 mg daily of ribavirin (minimum)
2. Dose adjustment is dependent on patient’s tolerance to the drug(s) and is at the discretion of the treating physician. (see H.1.d. and I.4.b.)

E.7.b. A positive HCV-RNA obtained at least 20 weeks after the start of the adequate course of treatment with pegylated interferon with ribavirin and before Screening for HALT-C. This test may be obtained either on or off treatment.

E.7.c. A pre-treatment liver biopsy which is available and has been performed within 18 months of randomization or a liver biopsy which is performed at least 8 weeks after the end of treatment and no more than 24 weeks prior to randomization. Slides from the biopsy must demonstrate at least Ishak stage 3 fibrosis as judged by the clinical center pathologist and then must be read centrally before randomization.

E. 7. d. Patients with a history of Ishak stage 3 or higher fibrosis, who subsequently have fibrosis scored as Ishak 2 can also be enrolled as Express patients. Both biopsies must be read by the local HALT-C pathologist using the Ishak fibrosis scoring system. The most recent Ishak fibrosis score will be used for determination of the study outcome.

E. 8. A willingness by all women of child bearing potential to utilize adequate contraception during the Main Trial study. Use of adequate contraception is not mandated during the extension trial since no medication is being offered.

E.9. A willingness by all men to utilize adequate contraception during the time they are treated with interferon-ribavirin combination therapy and for 6 months thereafter.

F. EXCLUSION CRITERIA

F.1. Liver histology which, in the opinion of the study pathologist, is consistent with: Any other co-existent cause of chronic liver disease defined as follows:

F.1.a. Hepatitis B surface antigen (HBsAg) positive within the past 12 months.

F.1.b Autoimmune hepatitis as defined by the following criteria:

1. A titer for anti-nuclear antibody of 1:160 or greater;
   and either 2 or 3:
2. Liver histology, in the opinion of the study pathologist, consistent with auto-immune hepatitis; and/or
3. Previous response to immunosuppressive therapy.

F.1.c. Autoimmune cholestatic liver disorders as defined by the presence of all of the following criteria:

1. A persistent elevation in serum alkaline phosphatase.
2. A titer for anti-nuclear or anti-mitochondrial antibodies of greater than 1:160 or 1:40 respectively.
3. Liver histology, in the opinion of the study pathologist, that is consistent with either primary biliary cirrhosis or sclerosing cholangitis.

F.1.d. Wilson's disease as defined by both of the following criteria:

1. A value for ceruloplasmin below the limits of normal.
2. Liver histology which, in the opinion of the study pathologist, is consistent with Wilson's disease.

F.1.e. Alpha-1-antitrypsin deficiency as defined by both of the following criteria:

1. A serum value for alpha-1-antitrypsin less than normal.
2. Liver histology which, in the opinion of the study pathologist, is consistent with alpha-1-antitrypsin deficiency.

F.1.f. Hemochromatosis or secondary iron overload as defined by 1 and 2 below:

1. An elevated value for serum ferritin or an iron saturation (serum iron/IBC x 100%) of greater than 50% and
2. Presence of 3+ or 4+ stainable iron on liver biopsy according to the study pathologist or a history of previous phlebotomy for iron overload.

All patients meeting the above criteria must undergo *HFE* genetic testing. Patients with an *HFE* genetic test demonstrating homozygosity for C282Y or compound heterozygosity, i.e. C282Y +/- and H63D -/+ are not eligible.

Patients who do not have the *HFE* genotypes just described may be entered into the study after first undergoing phlebotomy therapy to remove hepatic iron and then undergoing repeat liver biopsy demonstrating less than 3+ hepatic iron.

Any patients who have had HFE genetic test demonstrating homozygosity for C282Y or compound heterozygosity, i.e. C282Y +/- and H63D -/+ are not eligible.

F.1.g. Steatohepatitis (alcohol or non-alcoholic), defined as severe histologic changes to include <u>all</u> of the following 3 criteria:

1. Steatosis (marked);
2. Mallory bodies (many);
3. Zone 3 pericellular fibrosis (extensive).

F.1.h. Drug-induced liver disease

F.2. A Child-Turcotte-Pugh score of greater than or equal to 7 points or any history of ascites, or any history of hepatic encephalopathy, or current evidence of ascites as defined in Appendix A. See Appendix B for a definition of the CTP score and for an adjustment to this score for patients with Gilbert's syndrome.

F.3 Any documented history of bleeding from either esophageal or gastric varices.

F.4. A platelet count of less than 50,000/mm^3. Pre-treatment or screening platelet count values may be used by the Express Group for eligibility requirements.

F.5. A neutrophil count of less than 1,000/mm^3. Pre-treatment or screening neutrophil count values may be used by the Express Group for eligibility requirements.

F.6. A hematocrit of less than 33% or hemoglobin less than 11 gm/dL. Pre-treatment or screening hematocrit and hemoglobin values may be used by the Express Group for eligibility requirements.

F.7. An alpha-fetoprotein of greater than 200 ng/mL for Lead-in patients, and an AFP of greater than 1000 for Express patients.

F.8. Evidence of an hepatic mass lesion by either ultrasound, CT or MR scan that is suspicious for hepatocellular carcinoma.

F.9. Renal insufficiency defined by a serum creatinine greater than 1.5 mg/dL.

F.10. A positive test for HIV confirmed by Western blot obtained within the past 12 months. HIV testing will be performed using standard protocols for confidentiality and notification of health authorities. A standard separate consent HIV testing consent form will be used at all participating centers.

F.11. Diabetes that, in the opinion of the investigator, is not controlled by diet, an oral hypoglycemic agent, and/or insulin.

F.12 Patients with hemophilia.

F.13. Patients who have received an organ, limb or bone marrow transplant.

F.14. Patients who require the use of certain chronic medications such as immunosuppressive medications (corticosteroids, methotrexate, azathioprine) or coumadin should be off medications for six months or longer before being screened.

F.15. Patients with active systemic autoimmune disorders such as rheumatoid arthritis, systemic lupus, etc.

F.16. Patients who have had a malignancy diagnosed and/or treated within the past 5 years, except for localized squamous or basal cell cancers treated by local excision, and those who have been adequately treated and have an excellent chance of cancer-free survival, in the opinion of the oncologist**.**

F.17. Patients with serious cardiac, cerebrovascular or pulmonary disease that, in the opinion of the investigator would preclude treatment with interferon and/or ribavirin.

F.18. Patients with underlying hematologic abnormalities that, in the opinion of the investigator, would preclude treatment with interferon.

F.19. Patients with a history of seizure disorder that has not been well-controlled by anti-seizure medications within the past 2 years.

F.20. Women who are pregnant or breast feeding.

F.21. Male partners of women who are pregnant or breast feeding.

F.22. Patients with active alcohol abuse within the past 12 months.

F.23. Patients who have used illicit drugs (e.g., heroin, cocaine, angel dust, etc.) within the past 2 years.

F.24. Patients with a history of any one of the following:

Suicide attempt or hospitalization for depression within the past 5 years.

1. Any current (within 6 months) severe or poorly-controlled psychiatric disorder (e.g., depression, schizophrenia, bipolar illness, obsessive-compulsive disorder, severe anxiety, personality disorder).
2. The following patients must be assessed and followed (if recommended) by a psychiatrist or other mental health professional:
   a. Patients who have had a suicide attempt and/or hospitalization for depression more that 5 years ago.
   b. Patients who have had a severe or poorly-controlled psychiatric disorder (e.g., depression, schizophrenia, bipolar illness, obsessive-compulsive disorder, severe anxiety, personality disorder) more than 6 months ago but less than 5 years ago.

F.25. Patients who have been intolerant to previous interferon therapy.

F.26. Patients who are unable to provide informed consent.

F.27. Patients who are unable or unwilling to undergo three liver biopsies over 4 years for assessment of hepatic histology during this trial.

F.28. Patients with a serum bilirubin above 2.5 mg/dL, except for:

1. a patient with Gilbert's syndrome, in the opinion of the investigator or
2. an Express patient being treated with ribavirin.

F.29. Patients participating in another clinical trial can be screened 6 months after the end of study drug.

F.30. Any other condition which, in the opinion of the investigator, would make the subject unsuitable for enrollment, or could interfere with the subject participating in or completing the protocol.

F31. Lead-in patients: Documented virologic response (undetectable HCV-RNA) within 4 weeks prior to or 6 months after discontinuing the most recent adequate course of interferon.

F32. Patients who have undergone liver transplantation.

G. SCREENING OF PATIENTS

G.1. Identification of potential patients.

G.1.a. The medical records of patients with chronic hepatitis C who have been previously treated with interferon or interferon/ribavirin combination therapy, and who are being followed or have been recently referred to a HALT-C Trial Treatment Center will be reviewed.

G.1.b. Patients who satisfy all preliminary entry criteria and are not excluded may enter screening. Preliminary entry criteria include all of the following:

1. Prior treatment with Interferon for at least 12 weeks as defined in E.3. or E.3.a.
2. Non-response to this prior treatment as defined in E.4.

G.1.c. If a patient is ineligible as a result of a single aberrant laboratory value, then that value may be repeated within 4 weeks and used instead of the initial value.

Patients initially not meeting screening criteria or not completing the screening process or Lead-in Phase (for Lead-in Group) may be re-evaluated no more than twice at intervals of no less than 2 months. Express patients may not be re-screened. Week 20 Responders who subsequently are in the Breakthrough or Relapse Group may not be re-screened. Patients who are re-screened need to meet all inclusion and exclusion criteria. Screen 1 and screen 2 tests and procedures must be repeated, except the

Skinner Alcohol Questionnaire, the CIDI, the Block Food Questionnaire and collection of blood for EBV transformations. The previous ultrasound and biopsy, HBsAg and HIV results may be used if they were performed within the specified time windows.

G.1.d. Each HALT-C Trial Treatment Center will maintain a log of all patients screened for the HALT-C Trial.

G.1.e. Recent legislation has required that the NIH ensure that federally-funded clinical trials include sufficient numbers of women and designated minority groups to determine whether the intervention affects women or members of minority groups differently from other subjects. The goal for this trial is to recruit at least 20% from minority groups and 30% women.

G.1.f. Screening for the HALT-C Trial is expected to last for 2 years.

G.1.g. Confidentiality of subject data will be maintained throughout the trial. No subject identifiers will be used and all data entered into the trial database will be under code.

The HALT-C Trial has received a Certificate of Confidentiality from the Department of Health and Human Services (DHHS). This certificate protects the investigators from being forced to release any research data with patient identifiers, even under court order or subpoena, without written consent from a patient. This includes information collected on genetic testing and HIV status, excluding required notification of health authorities for HIV positive test results. This protection, however, does not prohibit the investigators from voluntarily reporting information about subject's threatened violence to self or others.

G.1.h. The Principal Investigator or Co-Investigator, or approved designee will obtain informed consent from patients after full review of the pros and cons of the study. The possible benefits and complications of participation will be explained in detail. Every effort will be made to ensure that the patient fully comprehends the nature of the study and the details of his or her participation. A copy of the consent form will be provided to the subject.

G.2. Screening of patients.

G.2.a. The specific aims and general conduct of the protocol will be reviewed with each potential patient.

G.2.b. If the patient wishes to be screened for possible inclusion in this study, he or she must then sign the screening consent form.

G.3. Screening visit #1:

The patient will then undergo the first of two screening visits that will include the following:

G.3.a. History and physical examination

G.3.b. Laboratory blood tests to include:

1. Serum chemistries (BUN, creatinine, glucose, uric acid, and triglycerides);
2. Liver chemistries (AST, ALT, alkaline phosphatase, total bilirubin, albumin, globulin (or total protein);
3. Complete blood count (WBC count and neutrophil count, hematocrit, hemoglobin and platelets);
4. Pro-thrombin time (INR);
5. Alpha-fetoprotein;
6. Serologic screening tests to exclude other causes of chronic liver disease if these tests were either not previously performed or the results can not be obtained (e.g., HBsAg, ANA, HIV, ferritin, serum iron, iron binding capacity);
7. HCV RNA (to be sent to Virology Core Laboratory);

G.3.c. Completion of the Composite International Diagnostic Interview (CIDI) questionnaire regarding active drug and alcohol use, anxiety and depression. Those sites that have special difficulties in obtaining the CIDI, will have the option of excluding this questionnaire from the Screening Visit.

G.3.d. Eligible subjects with a history of severe or dose-limiting neuropsychiatric toxicity during prior interferon treatment will be referred to a consulting psychiatrist/psychologist. In addition to clarifying possible psychiatric disorders, the consultant will help determine current suitability for this study and the need for prophylactic therapy such as anti-depressants or counseling.

G.4. Screening visit #2:

All patients who meet the study criteria for the first screening examination will then proceed to the second screening visit and undergo the following testing:

G.4.a. Laboratory blood tests to include:

1. Liver chemistries (AST, ALT, alkaline phosphatase, total bilirubin, albumin, globulin (or total protein);
2. Thyroid Stimulating Hormone
3. Whole blood will be sent to the repository for the isolation of PBMCs and generation of EBV transformed cell lines. Blood will be drawn for EBV cell lines only if the patient has provided consent for genetic testing.

G.4.b. Completion of questionnaires regarding life-long alcohol use, quality of life and Beck Depression Inventory.

G.4.c. Sign informed consent to enter the trial Lead-in Phase and/or Randomized Phase if not included in initial consent.

G.4.d. A urine specimen will be collected from women of child-bearing potential for a pregnancy test and from all patients for the presence of protein and heme by dipstick.

G.4.e. Performance of additional procedures as necessary to enter the study:

1. Liver biopsy, if not performed within the past 12 months.
2. Ultrasound examination of the liver if not performed within the past 6 months.
3. Biopsy specimens will be sent to the AFIP for central reading following evaluation by the clinical center HALT-C Trial Pathologist**.**

G.4.f. Ancillary Studies

1. Sign informed consent form for ancillary studies.
2. Perform additional testing necessary for ancillary studies.
3. Refer to section Q.4. for a list of Ancillary studies.

H. LEAD-IN PHASE:

All patients who meet and completely fulfill study criteria at both screening visits are eligible to enter the Lead-in Phase of this trial except eligible Express patients who will enter the Randomized Phase of this trial. (Section J)

H.1. Baseline visit:

H.1.a. The baseline visit should be held within 14 weeks of screening visit #1.

H.1.b. Baseline data will be obtained as shown in Table 1.

H.1.c. Instructions and study medications will be distributed to patients.

H.1.d. All patients who have neutrophils >1,500/mm^3 and platelet count >75,000/mm^3 will initially be treated for 24 weeks with peginterferon alfa-2a180 μg once weekly plus ribavirin 1000-1200 mg (prescribed according to weight <75kg, >75kg) daily in two divided doses. Patients previously intolerant to ribavirin may receive peginterferon alfa-2a alone, at the discretion of the site investigator.

H.1.e. Patients who are admitted to the study with platelet count from 50,000/mm^3 up to 75,000/mm^3 and/or neutrophils between 1,000/mm^3 to 1,500/mm^3, can be enrolled but the following special precautions will apply:

Safety measures for patients with platelet count under 75,000/mm^3 and/or neutrophils under 1,500/mm^3

1. The ribavirin dosage may be unchanged or lowered at the PI's discretion. Patients will start at a reduced dose of 90 μg of peginterferon alfa-2a once weekly.

2. Patients will be monitored more closely with the addition of a CBC with differential at Week 1 and Week 6.
3. Patients will be asked to hold weekly dose of peginterferon alfa-2a until the results of the CBC with differential are assessed by the PI.
4. The first 25 patients entering the Lead-in Phase with a lowered platelet and/or neutrophil count will have ongoing monitoring by the DSMB for the first 8 weeks of treatment.
5. A separate dose reduction scheme will be followed for these patients (see Appendix D, Table c).
6. Dose adjustment upwards of peginterferon alfa 2-a should be made at the discretion of the PI after 8 weeks in the Lead-in.

H.1.f. G-CSF or GM-CSF may not be used in the Lead-in Phase.

H.2. Weeks 2-20.

During this lead-in treatment phase all patients will be seen and examined at regular intervals, and will undergo various laboratory studies as defined in Table I.

I. CONTINUED FOLLOW-UP OF WEEK 20 RESPONDERS

I.1. Eligibility for continued treatment at week 24:

I.1.a. Patients who have a virologic response (undetectable HCV RNA as determined by the Central Virology lab) at week 20 will be treated through week 48 and follow-up through week 72. If virologic breakthrough or relapse is documented by detection of HCV RNA at weeks 36, 48, 60 or 72, patients will be considered for randomization (Section J). Any virologic breakthrough or relapse must be confirmed by a blood draw on a separate day. When a patient has had two detectable HCV RNA tests (as determined by the Central Virology lab) s/he will be offered enrollment into the Randomization Phase.

I.2. Treatment discontinuation:

Patients will be discontinued from treatment at Week 24 and at any point thereafter if they develop any of the criteria listed in Section L.

I.3. Patient Visits:

I.3.a. Will occur every 6 weeks until Week 48, as well as at Week 60 and Week 72.

I.3.b. Blood will be drawn and questionnaires will be completed at each visit according to schedule in Table 3.

I.4. Management:

I.4.a. Study drugs will be continued for a total of 48 weeks.

I.4.b. Medication management will be at the Principal Investigator's discretion.

J. RANDOMIZED PHASE

J.1. Assessment for Randomization

J.1.a. Patients who do not have a virologic response may be assessed for randomization at the following times:

1. between weeks 20 and 24 for those who have gone through the Lead-in Phase,
2. during Screening for the Express patients,
3. at weeks 36, 48, 60 or 72 for Week 20 Responders. Any virologic breakthrough or relapse must be confirmed by a blood draw on a separate day. Patient must be willing to be re-biopsied if their baseline (Screening) biopsy is greater than 30 months from the time of Breakthrough/Relapse Randomization.
4. After Week 72, a W20 Responder HALT-C patient who relapses may be re-screened as an Express patient.

J.1.b. The investigator will confirm the patient's eligibility for randomization, as follows:

1. A serum sample will be drawn and sent to the Virology Core Laboratory (via the Central Repository) for determination of HCV RNA.
2. AFP determination and Ultrasound examination of the liver will be performed for HCC Screening.
3. Laboratory tests, including liver chemistries, routine chemistries, CBC, TSH, and PT will be obtained.
4. Calculation of Child-Turcotte-Pugh score.
5. Willingness to participate in the trial for 4 years after randomization.

J.1.c. Patients with detectable HCV RNA at weeks 20, 36, 48, 60 or 72 and during Screening for Express patients will not be eligible for the Randomized Phase if they have any of the following:

1. elevation of serum alpha-fetoprotein greater than 1000 ng/mL
2. evidence of HCC on ultrasound
3. Child-Turcotte-Pugh score greater than or equal to 7 at 2 consecutive visits:
   - week 12 and week 20 for those patients entering through the Lead-in Phase
   - 2 separate Screening visits for Express patients
   - Weeks 36, 48, 60, 72 for Week 20 Responders
4. unwillingness to repeat a baseline (Screening) biopsy which is greater than 30 months from time of Breakthrough/Relapse Randomization. This biopsy must be read by the Central Pathology for stratification of Randomization.

Patients who discontinue peginterferon alfa-2a treatment during the Lead-in Phase or during treatment in the parallel responder protocol are not eligible to be randomized. (see L.3.)

J.1.d. Prior to randomization, patients will be stratified on the basis of the presence or absence of cirrhosis, as determined by central reading of baseline (Screening) biopsy. Breakthrough/Relpase patients will be stratified on the basis of the central reading of the most recent liver biopsy. Patients will not be excluded if the central reading of their biopsy is less than fibrosis stage 3

J.1.e. All eligible patients will be randomly assigned to one of two groups as follows:

1. To continue treatment with peginterferon alfa-2a alone at a dose of 90 μg administered once weekly for an additional 42 months.

   If a patient entering the Randomized Phase has been on 45μg peginterferon alfa-2a, a dose increase back to 90μg may be made at the Principal Investigator's discretion, but is not mandatory.
2. To stop peginterferon alfa-2a/ribavirin therapy and be followed prospectively for an additional 42 months without treatment.
3.

J.1.f. G-CSF or GM-CSF may not be used in the Randomized Phase.

J.2. Follow-up schedule:

All patients, in both arms of the study, will be seen at regular intervals, examined and undergo various laboratory studies as defined in Table 2. Results of these tests and procedures may be shared with the patient.

In the event that a patient who is randomized cannot/will not complete all of the scheduled study visits, the patient will be offered HCV-RNA testing, an ultrasound, a liver biopsy and endoscopy at their last study visit, if deemed appropriate by the PI, in addition to all regularly scheduled tests and procedures. Every effort will be made to continue collecting any data possible on all patients who have been randomized.

J.3. Liver biopsy:

All patients will undergo repeat liver biopsy at study visit month 24 and study visit month 48 regardless of treatment group.

Only percutaneous biopsies will be performed. Patients with a prothrombin time $>$ 1.5 INR or platelet count $<$ 50,000 will not be biopsied.

J.4. Post-treatment follow-up:

Patients in both groups will be followed, but not treated, for an additional 6 months after study visit Month 48 (Table 2, study visit Month 54). Additional follow-up visits will be offered to patients at Month 60 and Month 72 until April 2007. After April 2007 follow-up visits every six months will be offered to all patients who are being currently followed in the Main Trial except for those patients who have undergone liver

transplantation.

J.5. Endoscopy:

An endoscopy will be performed within 4 weeks of the randomization visit if the patient has not had an endoscopy performed within the past 12 months.

K. OUTCOME VARIABLES

K.1. Primary Outcome Variables

The major outcome variable used in this study will be progression of liver disease as judged by any of the following outcomes:

K.1.a. Increase in Ishak fibrosis score by 2 points or more at 2 or 4 year biopsies

K.1.b. Death from any cause

K.1.c. Development of HCC

K.1.d. CTP score of 7 or higher at two consecutive study visits

K.1.e. Variceal hemorrhage

K.1.f. Ascites

K.1.g. Spontaneous bacterial peritonitis

K.1.h. Hepatic encephalopathy

K.2. Secondary Outcome Variables

Secondary outcome variables will include:

K.2.a. Quality of life,

K.2.b. Serious adverse events, and

K.2.c. Events requiring dose reductions (in both treatment groups).

K.2.d. Changes in fibrosis from baseline at year 2 or year 4 biopsy.

K.2.e. Presumed Hepatocellular Carcinoma

L. CESSATION OF TREATMENT

L.1. Outcomes which require permanent discontinuation of treatment:

L.1.a. Death from any cause

L.1.b. Liver transplant

L.1.c. HCC

L.1.d. UNOS Status 2b as defined by the 1999 UNOS Transplant Criteria Meeting:

1. Presence of a small hepatocellular carcinoma; or
2. CTP score of 10 or more; or
3. CTP score of 7 or more plus any of the following:
   - Documented unresponsive variceal hemorrhage
   - Hepatorenal syndrome
   - Occurrence of one episode of spontaneous bacterial peritonitis
   - Refractory ascites unresponsive to treatment

L.2. By these criteria, onset of ascites, variceal hemorrhage, or hepatic encephalopathy do not require discontinuation of peginterferon alfa-2a but may lead to withholding further therapy if, in the opinion of the investigator, continuation is not in the patient's best interest.

L.3. Randomized patients who stop receiving peginterferon alfa-2a will continue to be followed at regular visit intervals for the duration of the trial (if possible). Patients who discontinue treatment during the Lead-in Phase will not be followed beyond 24 weeks except for follow-up on all unresolved adverse events.

M. DOSE MODIFICATION:

M.1. Peginterferon alfa-2a dosing:

M.1.a. Factors that will lead to a reduction in the dose of peginterferon alfa-2a include:

1. Disabling symptoms, which, in the opinion of the investigator, are related to peginterferon alfa-2a treatment and prevent the patient from performing his/her occupation or daily tasks.
2. A rash consistent with allergic reaction or vasculitis.
3. Reductions in the platelet count according to the guidelines in Appendix D4 (page 67).
4. A reduction in neutrophil count according to the guidelines in Appendix D4 (page 67).
5. Any adverse reaction, which, in the opinion of the investigator, places the patient at increased risk.

M.1.b. The dose of peginterferon alfa-2a may be reduced as follows:

There are 3 prescribed levels for dose reduction:

- 135 μg
- 90 μg
- 45 μg

During the randomized phase 90μg may be reduced to 45 μg for cytopenias or other side effects. Peginterferon alfa-2a can be increased to 90μg at the investigator's discretion.

Please refer to Appendix D4 (pages 65 - 70), peginterferon alfa-2a Dose Adjustment Guidelines for complete information on dose adjustments.

M.1.c. Once a patient's dose has been decreased, the investigator may attempt to increase the dose back to or toward the previous stable level only if the following conditions are satisfied:

1. the event or circumstance responsible for the dosage adjustment has resolved or improved;
2. the patient has been at the lower dose for ≤4 consecutive doses;
3. ≤6 total doses have been administered to the patient at the lower level during the entirety of the treatment period. As a guideline, patients who have received more than 4 consecutive or 6 total doses of peginterferon alfa-2a at the lower dose level should not have their dosage regimen readjusted upward. If 4 or more consecutive doses of peginterferon alfa-2a are held or otherwise not administered (i.e., the patient has not received test medication for more than 28 days), the patient should be considered intolerant of the test medication or non-compliant, whichever is more appropriate to the clinical situation. In such cases, the investigator should consider discontinuation of study medication.

M.1.d. Every attempt will be made to keep those patients randomized to treatment with peginterferon alfa-2a on therapy by dose reduction.

M.2. Ribavirin dosing:

M.2.a. The dose of ribavirin will be reduced during the Lead-in Phase as follows:

<u>If either of the following is confirmed</u>: (1) a patient *without* significant cardiovascular disease experiences a fall in hemoglobin to <10 g/dL and ≥8.5 g/dL or (2) a patient *with* stable cardiovascular disease experiences a fall in hemoglobin by ≥2 g/dL during any 4 weeks of treatment, the ribavirin dose should be reduced to 600 mg per day (200 mg in the morning and 400 mg in the evening). Further reductions may be considered.

Patients who have more than a 3 g/dL decrease from baseline in their hemoglobin concentration should have consideration given to an appropriate work-up for anemia, including reticulocyte count, search for sources of bleeding, etc., especially if a further drop occurs following some weeks of apparently stable hemoglobin levels on ribavirin.

M.2.b. If a patient cannot tolerate ribavirin, then he or she can be treated with peginterferon alfa-2a alone and randomized, providing eligibility criteria are met. Ribavirin should be discontinued under the following circumstances:

1. If a patient without significant cardiovascular disease experiences a fall in hemoglobin confirmed to be less than 8.5 g/dL.
2. If a patient with stable cardiovascular disease maintains a hemoglobin value <12 g/dL despite 4 weeks on a reduced dose [21].

In the event of ribavirin being discontinued, it can be reintroduced at a daily dose of 600 mg and increased thereafter at the investigator's discretion. In the event of ribavirin being reduced, it can be increased at the investigator's discretion. It is not considered necessary to tailor the reduced dosing according to the 75 kg cut-off level.

M.3. Pregnancy:

M.3.a. If a patient becomes pregnant during the Lead-in Phase, treatment will be stopped and she will not be eligible for the Randomized Phase of the trial.

M.3.b. If a patient becomes pregnant during the Randomized Phase, treatment will be discontinued for the duration of the pregnancy. Treatment may be resumed three months post-partum if the patient is not breast feeding.

M.3.c. If a male patient's partner(s) becomes pregnant during the Lead-in Phase, ribavirin will be stopped, but not peginterferon alfa-2a. The male patient and his pregnant partner will be advised to use a barrier method of contraception for the remainder of the pregnancy, and post-partum if the male patient's partner is breast feeding.

N. ADVERSE EVENTS

N.1. Definition

N.1.a. An adverse event is any adverse change from the patient's baseline (pre-treatment) condition, including intercurrent illness which occurs during the course of the trial, after the consent form has been signed, whether the event is considered related to treatment or not.

N.1.b. A serious adverse event is an untoward medical occurrence that results in any of the following:

1. Death
2. Is life threatening (risk of death at the time of the event).
3. Requires in-patient hospitalization or prolongation of existing
4. hospitalization
5. Results in persistent or significant disability/incapacity
6. Congenital abnormality or birth defect

Important medical events that do not result in one of the events listed above may be considered a serious adverse event when, based upon appropriate medical judgment, they may jeopardize the patient and may require medical or surgical intervention to prevent one of the outcomes listed above.

N.1.c. A serious adverse event which is unexpected and is drug related (even remotely) will require expedited reporting (see N.3.a.).

N.1.d. The following events are trial outcomes and will not be considered to be serious adverse events:

1. Development of HCC or presumed HCC
2. A CTP score of 7 or higher at 2 consecutive study visits

3. Ascites
4. Variceal hemorrhage
5. Hepatic encephalopathy
6. Spontaneous bacterial peritonitis

N.2. Data collection procedures for adverse events

N.2.a. At each follow-up visit through Month 54, patients will be interviewed regarding medical conditions, medical changes, and symptoms that have occurred since the last visit. An Adverse Event form will be completed if any adverse event is reported. If the Study Coordinator or Principal Investigator learns of any hospitalizations or other adverse events between study visits, an Adverse Event form will be completed. All adverse events and Serious Adverse Events as of May 1, 2007 will not be reported to the DCC.

N.2.b. A Serious Adverse Event form will be completed for all adverse events rated as serious.

N.2.c. Patients will be followed for all ongoing unresolved adverse events until they are either resolved, or in the opinion of the Principal Investigator, the patient is medically stable.

N.3. Reporting procedures

N.3.a. All serious adverse events, for patients in both the treated and the control group will be reported to the DCC and to Roche within 24 hours by telephone. The Serious Adverse Event form will be faxed to the DCC and Roche within 24-48 hours. This reporting includes serious adverse events that occur from the time the patient has signed the Screening or Trial informed consent to study visit Month 54 (6 month post Trial follow-up). Serious adverse events on patients who are in the control arm of the Randomized Phase and have stopped treatment for 12 weeks or patients who have not yet received treatment need to be reported only to the DCC.
Roche will review all serious adverse events and will notify the DCC if an event is considered to be unexpected. The DCC will distribute the expedited report to NIDDK, the FDA, the DSMB, and clinical centers.

Status reports on serious adverse events will be generated by the DCC and sent to the DSMB every month and will include the relationship of the adverse event to trial medications, the severity of the event and if the event is resolved or ongoing. Adverse events will be reported annually to the FDA, as required.

N.3.b. All deaths, in both the treated and in the control group will be reported to the DCC within 24 hours by telephone. The Death Report will be faxed to the DCC within 24 hours. This reporting begins at the time the patient has signed the Trial informed consent up to the last scheduled patient visit (through October 2009). The report will include the relationship of the death to trial medications. A Clinical Outcome form will also be completed and sent to DCC for distribution and review by the Outcome Review Board (ORB). Deaths will be reported immediately to NIDDK and the DSMB. A death

will be reported in an expedited report only if it is unexpected and drug related. A death must also be reported in accordance with local law and regulations.

O. SAMPLE SIZE

O.1. Estimates of rates for the primary outcome variable

O.1.a. The primary outcome variable for the trial will be the progression of liver disease as judged by either:

1. An increase in the Ishak fibrosis score of 2 points or more at the study_visit month 24 or study visit month 48 biopsies, or
2. The development of hepatic decompensation (as defined in K.1.), the development of HCC, or death from any cause.

O.2. Sample size estimate

O.2.a. The primary variable for this trial is the percent of patients whose liver disease has progressed (as defined in K.1.) at the time of the biopsy at study visit month 48. The sample size estimate is based on a chi-square test (171).

O.2.b. The null hypothesis is that the percent of patients with progression of liver disease will be the same in the peginterferon alfa-2a treated and control arms. The alternative hypothesis is that there will be at least a 50% reduction in the annual rate of progression in the peginterferon alfa-2a arm when compared to the control arm.

O.2.c. The sample size is based on a two-sided alpha (type I error) of 5% and a power of 90% (type II error).

O.2.d. If the annual rate of progression of liver disease is p, then the rate at the end of 3.5 years is calculated as:

$$1 - (1-p/2) * (1-p) * (1-p) * (1-p).$$

O.2.e. Noncompliance rates of 3%, 6%, and 9% have been assumed for the peginterferon alfa-2a arm in years 2, 3, and 4. These patients will continue to be followed and will experience progression at the rate of the control arm.

O.2.f. Similarly, it is assumed that 5%, 10% and 15% of the control arm will elect to be treated and will receive peginterferon alfa-2a in years 2, 3 and 4. These patients will continue to be followed and will experience progression at the rate of the peginterferon alfa-2a arm.

O.2.g. Under these assumptions, 18.7% of the control arm and 10.6% of the peginterferon alfa-2a arm will have had progression of their liver disease 3.5 years after randomization. A sample size of 810 patients (405 per arm) will have 90% power to detect this difference. Nine hundred patients will be randomized for this trial, allowing for a loss to follow-up of 90 patients.

O.3. Power Analyses

The following table shows the power for annual rates in the control arm of 5% and 6% and several noncompliance rates. It also shows the effect of assuming that the pre-randomization treatment protects the control arm for 0 months or 3 months.

Annual rate		Noncompliance rate (annual)		Lead-in	Rate at 3.5 years		
Control arm	PEG-IFN arm	Control arm	PEG-IFN arm	Control arm	Control arm	PEG-IFN arm	Power
6%	3%	0%	0%	0	19.4%	10.1%	96%
6%	3%	5%	3%	0	18.7%	10.6%	90%
6%	3%	10%	5%	0	17.9%	10.9%	80%
6%	3%	0%	0%	3 mos	18.8%	10.1%	94%
6%	3%	5%	3%	3 mos	18.0%	10.6%	86%
6%	3%	10%	5%	3 mos	17.2%	10.9%	73%
5%	2.5%	0%	0%	0	16.4%	8.5%	93%
5%	2.5%	5%	3%	0	15.7%	8.9%	84%
5%	2.5%	5%	3%	3 mos	15.2%	8.9%	79%

P. DATA MANAGEMENT AND ANALYSIS

P.1. Data Management

P.1.a. The DCC will provide a web-based data entry system. This system will be an adaptation of NERI’s Advanced Data Entry and Protocol Management System (ADEPT). Clinical Center staff will use this system for data entry of study forms. Information entered into the data entry system will be by patient study ID; names will not be linked with patient data in the database. Clinical centers will maintain records linking the patient name with the ID assigned for this trial in locked files.

The data entry system will include context specific help, automatic skip patterns, range checks, and intra- and inter-form checks of the data as it is being entered.

P.1.b. The system will produce visit schedules to assist the Clinical Center staff in the scheduling of appointments and visit control sheets that will list all of the forms and procedures for a scheduled visit. The system will also be able to produce lists of data as needed.

P.1.c. The data sets created by ADEPT will be converted to SAS data sets at the DCC. Additional data editing will be performed at the DCC using SAS. Outliers will be identified using predetermined limits and graphical methods. These edits will also compare patient data across forms and over time. Edit messages will be sent to the Clinical Centers for clarification as needed.

P.1.d. Summaries of each variable will be produced, including a histogram and distribution of all possible values for categorical variables, and scatterplots, means, medians, maxima, minima and standard deviations for continuous variables. The sources of missing data and the implications for analysis will be investigated.

P.2. Analysis of Baseline Data

P.2.a. For this trial, baseline data are collected prior to the lead-in or prior to the start of treatment for the Express patients. Some additional data are collected at the visit immediately preceding randomization.

P.2.b. The analyses of the baseline data will include Clinical Center comparisons, comparisons of patients who are randomized and those who are not, and comparison of the two randomized treatment groups. An analysis of missing data will also be prepared.

P.2.c. Baseline characteristics that form continuous variables will be compared using analysis of variance or t-tests. Data will be transformed (e.g. logarithms) prior to analysis if transformation is needed to meet the assumptions of normality and homogeneity of variances that underlie these methods. Non-parametric methods, such as the Wilcoxon test, will be used if data fail to meet these assumptions even after transformation (172).

P.2.d. Characteristics that form ordinal categorical variables, (e.g., Child-Turcotte-Pugh score, biopsy severity scores) may be compared using analysis of variance or t-tests if the number of categories is large enough for the data to approximately fit the assumption of normality. Otherwise non-parametric methods or methods for categorical data will be used. Categorical variables (e.g., presence or absence of cirrhosis) will be analyzed using chi-square tests (173,174).

P.2.e. It is expected, due to randomization, that the patients in the treatment arm will, on average, be similar with respect to variables that might influence outcome. However, for imbalances that persist despite randomization, co-variates or strata will be included in the analysis of the primary and secondary endpoints.

P.3. Analyses of Lead-in Phase

P.3.a. The primary outcome for the Lead-in Phase is the disappearance of HCV RNA. Logistic regression will be used to determine the characteristics that are predictive of a response to peginterferon alfa-2a and ribavirin.

P.4. Analyses of the Primary Outcome

P.4.a. All randomized patients will be included in the analysis of the primary outcome. This includes patients who are later found to be ineligible and patients who do not receive the assigned treatment.

P.4.b. The primary outcome of this trial is progression of liver disease as defined in Section K.

P.4.c. The analysis of this outcome is complicated because this is a composite outcome that consists of both the results of biopsies obtained at two-year intervals and the occurrence of events that may occur at any time during the follow-up period. In addition, the biopsy outcome, the development of cirrhosis, can occur only in patients in stratum 1 (pre-cirrhotic). Patients in stratum 2 will be much more likely to develop clinical events than patients in stratum 1.

P.4.d. If missing data are minimal, then the percent of patients having an outcome at the end of four years can be compared using logistic regression. This method will have less power than methods based on survival analysis. Several methods have been proposed for the analysis of interval-censored survival data (175-178). The method to be used will be described in detail in the Manual of Operations (MOO).

P.4.e. Hepatic events will be evaluated using life table methods and proportional hazards regression (45). The primary analysis will be life table analyses of time to first event. Life table estimates of time to hepatic event will be calculated for the two treatment groups with clinical center and baseline presence or absence of cirrhosis as strata. The log rank test will be used to test the difference between the treatment groups.

P.4.f. Analyses will be conducted for all hepatic events and the subset of death, HCC, or UNOS status 2b as defined in 1999.

P.5. Interim analyses

P.5.a. An interim analysis will be performed once 50% of the study visit Month 24 biopsies have been evaluated by the Central Pathology Committee. We recommend implementation of an early stopping rule based on an O'Brien-Fleming group sequential plan (179). This will be implemented using the alpha spending approach of Lan and DeMets (180-181).

P.5.b. Once the early stopping rule is established, NERI will produce interim analyses for the DSMB that will include:

1. Standard recruitment graphs comparing actual to goal over time and projected completion point.
2. Detailed tables and figures of recruitment experience at each CC (Number, by month or quarter by CC, as well as gender, age and minority cross-tabulations by CC).
3. Tables comparing baseline characteristics, cumulatively, at each interim analysis by (blindly labeled) treatment groups. These tables will be presented in aggregate as well as by CC.
4. Protocol and eligibility violations in trial patients will be reported since the last interim analysis and cumulatively, by CC with full documentation.

P.5.c. It is anticipated that the proposed stopping rule will have asymmetric boundaries to reflect the overriding concern with minimizing the possible deleterious

effect of peginterferon alfa-2a (if observed). We also strongly advocate maintaining, as long as feasible, blindness of the DSMB to actual treatment group, by arbitrary labels such as A and B. This provides additional security if copies of reports are seen by others and may assist the DSMB members in maintaining objectivity.

P.6. Analyses of Secondary Endpoints

P.6.a. Longitudinal methods will be used to analyze changes in quality of life and other measured variables (182).

Q. POLICIES AND PROCEDURES

Q.1. Trial Organization

The following are key components in this multi-site trial:

- Project Officer (NIDDK);
- The funded components: Clinical Centers, Data Coordinating Center, Virology Lab, Central Repository;
- Steering Committee (SC) (and subcommittees); and
- Data and Safety Monitoring Board.

Each of these components are discussed below. Figure 2 summarizes the main lines of communication between them.

Q.1.a. NIDDK

The Project Officer for the HALT-C Trial will be responsible for the close coordination of all aspects of the trial. All technical direction for the trial resides with the Project Officer who will serve as executive secretary of the Data and Safety Monitoring Board (DSMB) and will serve as a liaison between the DCC and the DSMB. The Project Officer will assist in quality control, interim data and safety monitoring, final data analysis and interpretation, preparation of publications, and coordination and performance monitoring. A representative of NIDDK will serve on each committee.

Q.1.b. Clinical Centers (CC)

Each clinical center will need to recruit as many as 180 patients and randomize and follow approximately 90 patients. Clinical Centers will be responsible for collecting trial data, recording information on the trial forms, sending these data to the DCC, and collecting and shipping specimens to the Repository.
Key Clinical Center responsibilities include:

- Recruiting adequate numbers of women and designated minority patients
- Providing bilingual interviews as needed
- Assisting with Phase 1 protocol development by serving on subcommittees, pre-testing sections of the protocol as required and taking appropriate leadership roles for protocol segments
- Completing recruitment on time
- Working proactively to retain patients and keep high adherence to therapy

- Completing all trial protocols according to the MOO and collaborating with the DCC to ensure high quality data
- Participating in manuscripts and presentations.

Q.1.c. Data Coordinating Center (DCC)

The DCC shall have primary responsibility for the following tasks:

- Preparation of the trial protocol, forms, data management system, and a management plan
- Monitoring and support of enrollment and follow-up
- Compilation and preparation of the data, assuring data quality, and data analysis
- Administrative support of the work of the Project Officer and the trial subcommittees
- Participation in manuscripts and presentations
- Developing and monitoring all Quality Assurance/Quality Control procedures

Q.1.d. Virology Laboratory (VL)

This central laboratory will perform key assays for the trial. Key responsibilities include:

- Participation in protocol development
- Leadership in development of the specimen processing and analysis protocols and production of the laboratory
- Completion of all assays in a timely manner
- Implementation of adequate quality assurance in assay performance
- Participation in manuscripts and presentations

Q.1.e. Central Repository (CR)

SeraCare Bio Services, formerly BBI-Biotech Research Laboratories, Inc, will serve as the Central Repository for the trial. This repository will be responsible for receipt, storage and distribution of serum, plasma, peripheral blood mononuclear cells (PBMC) and frozen liver tissue.

Specimens will be sent to the repository for indefinite storage. In the initial treatment phase, blood and tissue will be collected and white blood cells will be used for the generation of cell lines as well as for the extraction of DNA. These materials will be stored for genetic testing. Genetic tests will be confined to testing for genes relating to viral hepatitis and other liver diseases. It is possible that as new genes are identified that may be related to immune response to chronic viral infections such as hepatitis or other liver diseases or liver cancer, patient's DNA material will be used for such testing in order to improve our knowledge of the factors that influence disease progression in patients with hepatitis C.

Blood, cell lines, and tissue will be stored under a unique identifier, which can be traced back to the patient. If there is a medical reason to seek specific information from the patient in the future, patients will be notified and if appropriate, genetic counseling will be offered.

Q.1.f. Steering Committee (SC)

The SC is the primary decision making body for the trial, subject to NIDDK final approval.

1. Membership includes:

   NIDDK: PO (1 Vote)
   CCs: PI of each CC (1 Vote each)
   VL: PI of VL (1 Vote)
   DCC PI of DCC (1 Vote)

2. Chair: A chair of the SC is appointed on a rotating annual basis from CC PI's.

Q.1.g. Executive Committee

The Chair, PO, Scientific Advisor and DCC PI form a small Executive Committee that convenes weekly or bi-weekly with the following non-decision-making functions:

- Development of SC agendas
- Calling ad hoc SC meetings/calls if needed
- Follow-up on all SC decisions to ensure implementation
- Monitoring activities of SC Subcommittees
- Monitoring of recruitment and retention

Q.1.h. Subcommittees of the Steering Committee

In Phase 1, Subcommittees are focused on protocol development. In Phases 2 and 3, the focus is on standard implementation, quality control, analysis and manuscripts. Phase I Subcommittees include:

- Protocol
- Clinical Assessment
- Ancillary Studies
- Forms
- Pathology

Phase 2 & 3 Subcommittees may include:

- Recruitment and Retention Monitoring/Support
- Ancillary Studies
- Publications
- Quality Assurance
- Pathology
- Laboratory/Repository
- Clinical Outcomes (including Adverse Events)
- Exemption Committee
- Genetics Committee

Q.1.i. Data and Safety Monitoring Board

The primary purpose of this Board will be to monitor and provide independent ethical oversight for the trial. The DSMB will provide advice to NIDDK, as needed. This board will review the protocol developed during Phase 1, plans for recruitment and follow-up, and any other questions pertinent to the ethical conduct of the trial. It will review protocol changes and might suggest changes as needed. During Phase 2, it will monitor the data at regular intervals to determine whether significant benefit or harm has been demonstrated in either treatment group or whether there is other compelling need to stop the trial.

If the DSMB finds the treatment to be effective all patients enrolled into HALT-C will be notified. However, funds are not currently available to provide treatment to all study participants. Every effort will be made to assist all patients in obtaining treatment with peginterferon alfa-2a.

During the extension, a DSMB will continue to monitor the study.

The Principal Investigator of the DCC, the Project Officer, and at least one scientific advisor from NIDDK may participate as ex-officio, not-voting members of this board.

Q.1.j. Outcome Review Board (ORB)

An Outcome Review Board will be appointed to review all outcome events (Section K). Members of the Board will include Principal Investigators from the Clinical Centers. Clinical Centers will submit to the DCC the appropriate forms and source documents. Copies of these documents will be sent to two members of the ORB, and to a third if there is not consensus. Outcomes will be evaluated by the ORB based on pre-determined criteria for each outcome variable. ORB members will not know the treatment status of patients.

Q.2. Changes to the Protocol

The Steering Committee, NIDDK, clinical center IRB's and the DSMB must approve all changes to the protocol.

Q.3. Publication Policies

A Publications Committee will review all publications. No trial data may be presented or published without prior approval of the Publications Committee. Detailed publication policies will be included in the Manual of Operations.

Q.4. Ancillary Studies:

The Ancillary Studies Committee recommends and the Steering Committee approves all ancillary studies in the HALT-C Trial. The ancillary study policy and detailed protocols are in the HALT-C Manual of Operations.

TABLES AND FIGURES

Figure 1: Design of the HALT-C Trial

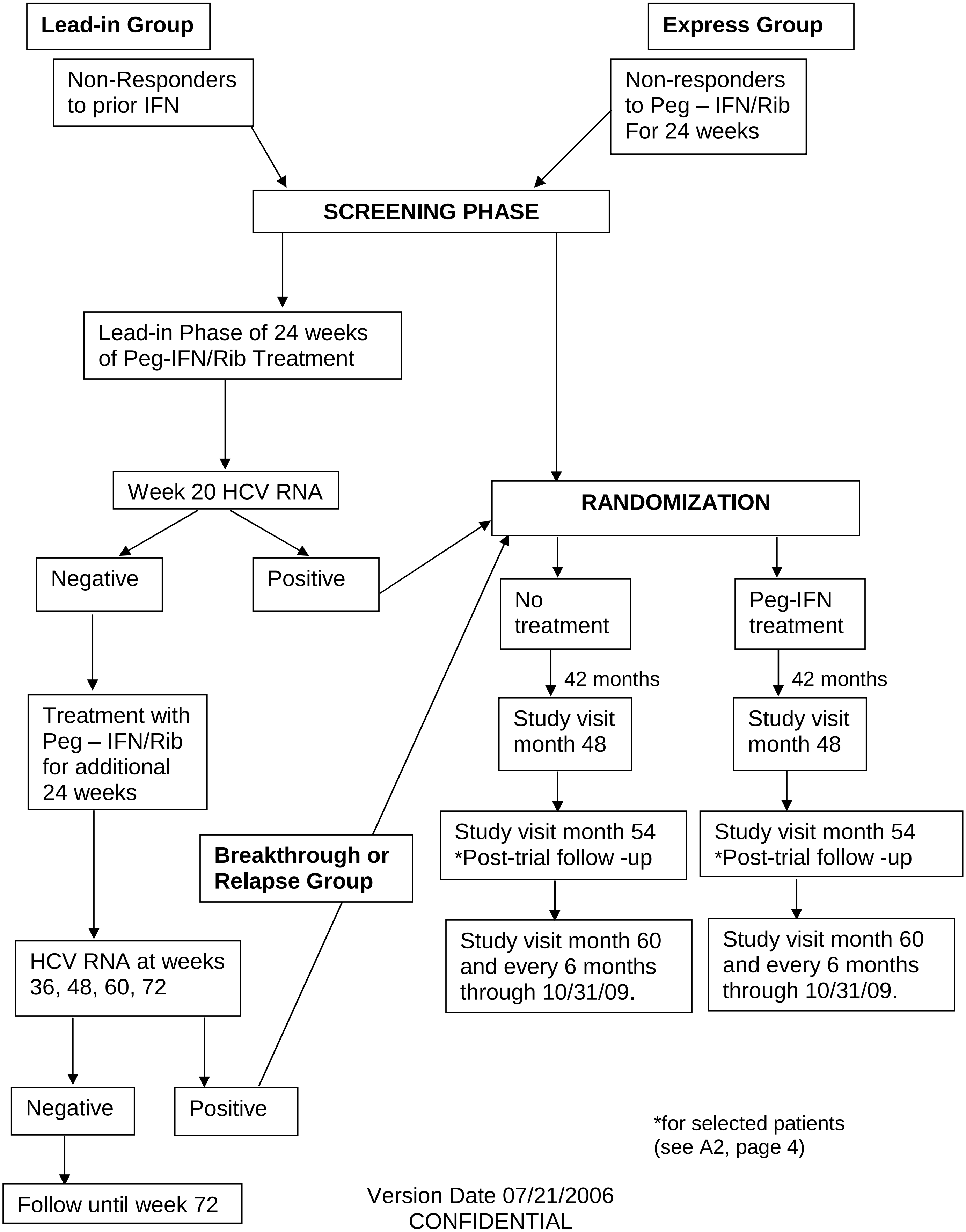

*for selected patients (see A2, page 4)

Figure 2: Trial Organization

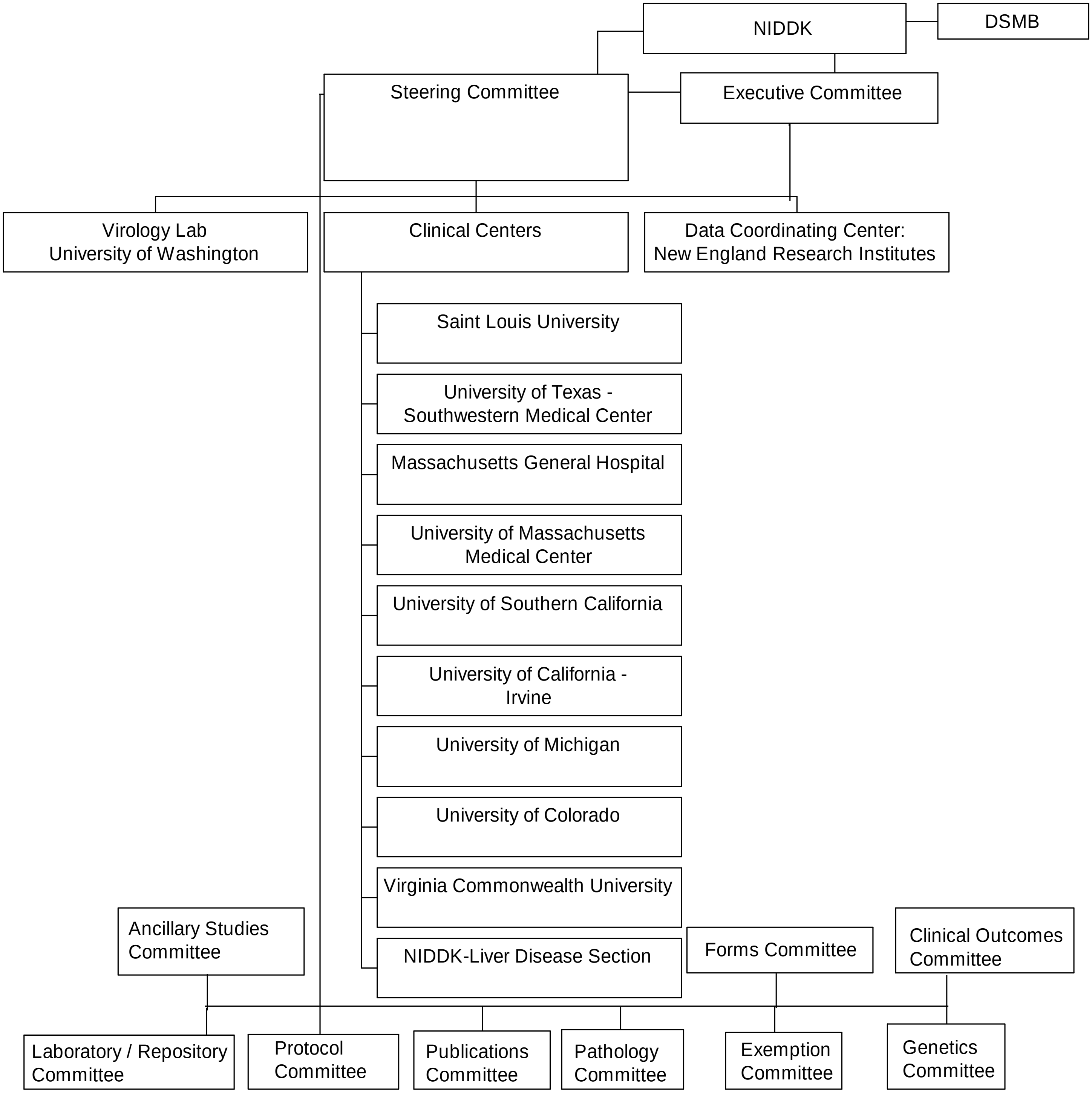

Table 1: Trial Flow Chart – Screening & Lead-in Phase (Lead-in Group)

Time	PE	LFT 4	Fast Chem (5)	CBC 6	Uric Acid	PT	AFP	TSH	U/A	Preg	HCV RNA	HIV	EBV	PBMC	U/S	Endo	L Bx	QOL	CIDI	ETOH Ques.	Skinner	Sx	Beck	Life Events
**SCREENING:**																								
Screen 1 (7)	X	X	X	X	X	X	X				X	X							X					
Screen 2		X						X	X	X			X	X	(1)		(3)	X			X	X	X	
**LEAD IN TREATMENT PEGINTERFERON alfa-2a PLUS RIBAVIRIN:**																								
Baseline		X	X	X	X	X	X			X	X									X		X		X
Week 2				X																				
Week 4		X		X						X														
Week 8		X		X						X												X		
Week 12	X	X		X		X		X		X	X												X	
Week 16		X		X						X														
Week 20	X	X	X	X	X	X	X	X		X	X				X							X	X	
Week 24		X		X						X	X					(2)				X				

(1). To be performed only in those patients who have not had an ultrasound within the previous 6 months.
(2). To be performed only in those patients who have not had an upper endoscopy within the previous 12 months. To be performed within 4 weeks of randomization visit.
(3). To be performed only in those patients who have not had a liver biopsy within the previous 12 months. Express patients must have had pre-treatment biopsy which is within 18 months of randomization.
(4). Liver Function Tests to include: AST, ALT, Alkaline Phosphatase, Total bilirubin, Total protein or globulin, Albumin.
(5). Fasting Chemistries to include: BUN, creatinine, glucose, triglycerides.
(6). CBC with differential and platelets.
(7). Serologic assays to include HBsAg and ANA if previous results of these assays are not available.

Table 2: Trial Flow Chart – Randomized Phase

TIME	PE	LFT	Fast Chem	CBC	Uric acid	PT	AFP	TSH	U/A	Preg	HCV RNA	HIV	DNA	PBMC	U/S	Endo	L Bx	QOL	ETOH Ques.	Sx	Beck	Life events
Month 9	X	X		X		X	X			X			X							X	X	
Month 12	X	X	X	X		X	X	X	X	X	X				X			X	X	X	X	X
Month 15	X	X		X		X	X						X							X	X	
Month 18	X	X	X	X		X	X	X			X		X						X	X	X	
Month 21	X	X		X		X	X							X (5)						X	X	
Month 24	X	X	X	X		X	X	X	X		X				X	X (4)	X	X	X	X	X	X
Month 27	X	X		X		X	X						X							X	X	
Month 30	X	X	X	X		X	X	X			X								X	X	X	
Month 33	X	X		X		X	X													X	X	
Month 36	X	X	X	X		X	X	X	X		X				X			X	X	X	X	X
Month 39	X	X		X		X	X													X	X	
Month 42	X	X	X	X		X	X	X			X								X	X	X	
Month 45	X	X		X		X	X							X (5)						X	X	
Month 48	X	X	X	X		X	X	X	X		X	X			X	X	X	X	X	X	X	X
Month 54	X	X		X		X	X				X							X		X	X	X
Month 60	X	X	creatinine	X		X	X				X (6)				X							
Month 72	X	X	creatinine	X		X	X				X (6)				X							

(1). Liver Function Tests to include: AST, ALT, Alkaline Phosphatase, Total bilirubin, Total protein or globulin, Albumin.
(2). Fasting Chemistries to include: BUN, creatinine, glucose, triglycerides.
(3). CBC with differential and platelets.
(4). To be performed on patients for whom varices were detected on the randomization Endoscopy.
(5). To be performed on a select group of patients.
(6). 2-4 serum aliquots for stored samples at the Repository for HCV-RNA or HCC markers.

Table 3: Trial Flow Chart – Week 20 Responders

TIME	PE	LFT (1)	FAST. CHEM (2)	CBC (3)	Uric acid	PT	AFP	TSH	U/A	Preg.	HCV RNA	DNA	U/S	Endo	LBx	QOL	ETOH ques	Symptoms	Beck	Life Events
Week 30		X		X						X		X						X		
Week 36	X	X		X		X	X	X		X	X^R	X	(4)					X	X	
Week 42		X		X						X		X						X		
Week 48	X	X	X	X	X	X	X	X	X	X	X^R		X				X	X	X	
Week 60		X	X	X		X	X				X^R	X	(4)					X	X	
Week 72	X	X	X	X		X	X	X			X^R	X	X			X	X	X	X	X
<u>BREAKTHROUGH or RELAPSE</u>																				
<u>R00 Week 24</u>		X	X	X	X	X				X	X			X			X	X	X	
<u>Turn to Table 2 Randomized Phase. Next visit will be Study Visit Month 9 of Randomized Phase.</u>																				

(1). Liver Function Tests to include: AST, ALT, Alkaline Phosphatase, Total bilirubin, Total protein or globulin, Albumin.
(2). Fasting Chemistries to include: BUN, creatinine, glucose, triglycerides.
(3). CBC with differential and platelets.
(R). Repeat confirmatory HCV RNA will be performed for those patients to be randomized.
(4). Only if randomized at Week 36 or Week 60. Ultrasound needs to be done before R00 visit.

Table 4: Trial Flow Chart – Screening & Randomization Visit (Express Group)

Time	P E	L F T (3)	Fast Chem (4)	C B C 5	Uric Acid	P T	A F P	T S H	U/A	Preg	HCV RNA	H I V	E B V	P B M C	U/ S	Endo	L Bx	Q O L	C I D I	ETOH Ques.	Skin-ner	S x	Beck	Life Events
Historical		X	X	X		X	X				X						(2)							
Screen 1 (67)	X	X	X	X	X	X	X				X	X							X					
Screen 2		X				X		X	X	X			X	X	(1)			X			X	X	X	
**RANDOMIZATION VISIT:**																								
R00 Randomi-zation Visit		X	X	X	X	X	X			X	X					X				X		X		X
Turn to Table 2 Randomized Phase. Next visit will be Study Visit Month 9 of Randomized Phase.																								

(1). To be performed only in those patients who have not had an ultrasound within the previous 6 months.
(2). Express patients must have had pre-treatment biopsy which is within 18 months of randomization.
(3). Liver Function Tests to include: AST, ALT, Alkaline Phosphatase, Total bilirubin, Total protein or globulin, Albumin.
(4). Fasting Chemistries to include: BUN, creatinine, glucose, triglycerides.
(5). CBC with differential and platelets.
(67). Serologic assays to include HBsAg and ANA if previous results of these assays are not available.

Table 5: Trial Flow Chart –Extension of the HALT-C Trial

TIME	P E	L F T	Fast Chem	C B C	Uric acid	PT	A F P	T S H	U/A	Preg	HCV RNA	H I V	D N A	PBMC	U/S	Stored serum	Q O L	ETOH Ques.	Sx	Beck	Life events
Month 54	X	X		X		X	X				X						X		X	X	X
Month 60	X	X	creati nine	X		X	X								X	X (1)					
Month 66	X	X	creati nine	X		X	X								X	X (1)					
Month 72	X	X	creati nine	X		X	X								X	X (1)					
Month 78	X	X	creati nine	X		X	X								X	X (1)					
Month 84	X	X	creati nine	X		X	X								X	X (1)					
Month 90	X	X	creati nine	X		X	X								X	X (1)					
Month 96	X	X	creati nine	X		X	X								X	X (1)					
Month 102	X	X	creati nine	X		X	X								X	X (1)					
Month 108	X	X	creati nine	X		X	X								X	X (1)					

(1) serum aliquots for stored samples

REFERENCES

1. Alter MJ. Epidemiology of Hepatitis C. *Hepatology* 1997;26:62S-5S
2. Ebeling F. Epidemiology of the Hepatitis C Virus. *Vox Sang* 1998;74:143-6
3. Hoofnagle JH. Hepatitis C: The clinical spectrum of disease. *Hepatology* 1997;26:15S-20S
4. Di Bisceglie AM. Hepatitis C and hepatocellular carcinoma. *Hepatology* 1997;26:34S-38S
5. Di Bisceglie AM. Hepatitis C and hepatocellular carcinoma. *Semin Liver Dis* 1995;15:64-69
6. Hassan F, Jeffers LJ, De Medina M, Reddy KR, et al. Hepatitis C-associated hepatocellular carcinoma. *Hepatology* 1990;12: 589-91
7. Yu MC, Yuan J, Ross RK, Govindarajan S. Presence of antibodies to the hepatitis B surface antigen is associated with an excess risk for hepatocellular carcinoma among non-Asians in Los Angeles County, California. *Hepatology* 1990;25:226-8
8. Di Bisceglie AM, Order SE, Klein JL, Waggoner JG et al. The role of chronic viral hepatitis in hepatocellular carcinoma in United States. *Am J Gastroenterol* 1991;86:335-8
9. De Bac C, Stroffolini T, Gaeta GB, Taliani G, et al. Pathogenic factors in cirrhosis with and without hepatocellular carcinoma: a multicenter Italian study. *Hepatology* 1994;20:1225-30
10. Koff RS, Dienstag JL. Extrahepatic manifestations of hepatitis C and the association with alcoholic liver disease. *Semin Liver Dis* 1995;15:101-9
11. Seeff LB: The natural history of chronic hepatitis C virus infection. *Clin in Liv Dis* 1997;1: 602
12. Tong MJ, El-Farra NS, Reikes AR, Co RL. Clinical outcomes after transfusion-associated hepatitis C. *N Engl J Med* 1995; 332:1463-6
13. Seeff LB, Buskell-Bales Z, Wright EC, Durako SJ, et al. Long-term mortality after transfusion-associated non-A, non-B hepatitis. *N Engl J Med* 1992; 327:1906-11
14. Di Bisceglie AM, Goodman ZD, Ishak KG, Hoofnagle JH, et al. Long-term clinical and histological follow-up of chronic posttransfusion hepatitis. *Hepatology* 1991;14:969-74
15. Takahashi M, Yamada G, Miyamoto R, Doi T, et al. Natural course of chronic hepatitis C. *Am J Gastroenterol* 1993;88:240-3
16. Jago JR, Gibson CJ, Diffey BL. Evaluation of subjective assessment of liver function from radionuclide images. *Br J Radiol* 1984;57:23-27
17. Hoefs JC, Wang F, Kanel G. Functional measurement of nonfibrotic hepatic mass in cirrhotic patients. *Am J Gastroenterol* 1997;92:2054-58
18. Hoefs JC, Green G, Reynolds TB, et al. Mechanism for the abnormal liver scan in acute alcoholic liver injury. *Am J Gastroenterol* 1984;79:950-8
19. Hoefs JC, Wang FW, Walker B, Kanel G: A novel, simple method of functional spleen volume calculation by liver-spleen scan. *J Nuc Med*, Accepted in March, 1999
20. Poynard T. Bedossa P, Opolon P. Natural history of liver fibrosis progression in patients with chronic hepatitis C. *Lancet* 1997; 349:825-32
21. Yano M, Kumada H, Hage M, Ikeda K, et al. The long-term pathological evolution of chronic hepatitis C. *Hepatology* 1996;23:1334-40
22. Vaquer P, Canet R, Llompart A, Riera J, et al. Histological evolution of chronic hepatitis C. Factors related to progression. *Liver* 1994;14:265-9
23. Seeff Leonard B. Natural history of hepatitis C. *Hepatology* 1997;26:21S-28S
24. Bosch J. Medical treatment of portal hypertension. *Digestion* 1998; 59: 547-55
25. Roberts LR, Kamath PS. Pathophysiology of variceal bleeding. *Gasteointest Endosc N Am* 1999: 9: 167-74
26. Hoefs JC, Jonas FM, Sarfeh IJ. Diagnosis and hemodynamic assessment of portal hypertension. *Surg Clin North Am* 1990; 70: 267-89
27. North Italian endoscopic club for the study and treatment of esophageal varices. Prediction of the first variceal hemorrhage in patients with cirrhosis of liver and esophageal varices: a perspective multicenter study. *N Engl J Med* 1988; 319; 983

28. Horisawa M, Goldstein G, Waxman A, Reynolds T. The abnormal hepatic scan of chronic liver disease: Its relationship to hepatic hemodynamics and colloid extraction. *Gastroenterology* 1976;71:210-213
29. Hoefs JC. Wang F, Kanel G, et al. The perfused Kupffer cell mass: Correlation with histology and severity of CLD. *Dig Dis Sci* 1995;20:552-60
30. Kodama T, Watanabe K, Hoshi H, Jinnouchi S, et al. Diagnosis of diffuse hepatocellular diseases during SPECT. *J Nucl Med* 1986;27:616-9
31. Gines P, Quintero E, Arroyo V, Teres J, et al. Compensated cirrhosis: Natural history and prognostic factors. *Hepatology* 1987;7:122-8
32. D'Amico G, Morabito A, Pagliaro L, et al. Survival and prognostic indicators in compensated and decompensated cirrhosis. *Dig Dis Sci* 1986;31:468-75
33. Magliocchetti N, Torchio P, Corrao G, Arico S, et al. Prognostic factors for long-term survival in cirrhotic patients after the first episode of liver decompensation. *Ital J Gastroenterol Hepatol* 1997;29:38-46
34. Navasa M, Rimola A, Rodes J. Bacterial infections in liver disease. *Semin Liver Dis* 1997; 17: 323-33
35. Runyon BA. Care of patients with ascites. *N Engl J Med* 1994; Fed 3: 330:337-42
36. Gines P, Sort P. Patholophysiology of renal dysfunction in cirrhosis. *Digestion* 1998;59: 11-15
37. Herrera JL. Management of acute liver failure. *Dig Dis* 1998; 16:274-83
38. Butterworth RF. Pathogenesis of acute hepatic encephalopathy. *Digestion* 1998; 59:16-21
39. Hoefs JC, Runyon BA: Spontaneous bacterial peritonitis. *Dis Mon* 1985;31:1-48
40. Collier J, Sherman M. Screening for hepatocellular carcinoma. Hepatology 1998; 23: 273-8
41. Castellano L, Calandra M, Del Vecchio Planco C, de Sio I. Predictive factors of survival and intrahepatic recurrence of hepatocellular carcinoma in cirrhosis after percutaneous ethanol injection: analysis of 71 patients. *J Hepatol* 1997; 27:862-70
42. Ikai I: Lymph node metastasis: proposal as a standard therapy. *Ann Surg* 1998; 227:443-9
43. Christensen E, Schlichting P, Fauerholdt L, et al. Changes of laboratory variables with time in cirrhosis: Prognostic and therapeutic significance. *Hepatology* 1985;5:843-53
44. Christensen E, Schlichting P, Fauerholdt L, et al. Prognostic value of Child-Turcotte criteria in medically treated cirrhosis. *Hepatology* 1984;4:430-5
45. Cox DR. Regression models and life tables. *J R Stat Soc* 1972;34:187-220
46. Christensen E. Multivariate survival analysis using Cox's regression model. *Hepatology* 1987;7:1346-58
47. Pugh RNH, Murray-Lyon IM, Dawson JL, et al. Transection of the esophagus for bleeding esophageal varices. *Br J Surg* 1973;60:646-64
48. Pasualetti P, Di Lauro G, Festuccia V, Giandomenico G, et al. Prognostic value of Pugh's modification of Child-Turcotte classification in patients with cirrhosis of the liver. *Panminerva Med* 1992;34:65-8
49. Reisman Y, Gips CH, Lavelle SM. Assessment of liver cirrhosis severity in 1015 patients of the Euricterus database with Campbel-Child, Pugh-Child and with ascites and ascites-nutritional state (ANS) related classifications. Euricterus Project Management Group. *Hepatogastroenterology* 1997;44:1376-84
50. Merkel C, Bolognesi M, Finucci GF, Angeli P, et al. Indocyanine green intrinsic hepatic clearance as a prognostic index of survival in patients with cirrhosis. *J Hepatol* 1989;9:16-22
51. Oellerich M, Burdelski M, Lautz HU, Schulz M, et al. Lidocaine metabolite as a measure of liver function in patients with cirrhosis. *Ther Drug Monit* 1990;12:219-26
52. Moreli A, Narducci F, Pelli MA, Farroni F, et al. The relationship between aminopyrine breath test and severity of liver disease in cirrhosis. *Am J Gastroenterol* 1981;76:110-3
53. Finucci G, Bellon S, Merkel C, Mormino P, et al. Evaluation of splanchnic angiography as a prognostic index of survival in patients with cirrhosis. *Scand J Gastroenterol* 1991;91:951-60

54. Vorobioff J, Groszmann RJ, Picabea E, Gamen M, et al. Prognostic value of hepatic venous pressure gradient measurements in alcoholic cirrhosis: a 10-year prospective study. *Gastroenterology* 1996;11:701-9
55. Merkel C, Gatta A, Zoli M, Bolognesi M, et al. Prognostic value of galactose elimination capacity, aminopyrine breath test, and ICG clearance in patients with cirrhosis. Comparison with the Pugh score. *Dig Dis Sci* 1991;31:1197-203
56. Katsuyoshi I, Mitchell D, Hann HL, Outwater EK, et al. Progressive viral-induced cirrhosis: Serial MR imaging findings and clinical correlation. *Radiology* 1998;207:729-35
57. Taylor KJW, Gorelick FS, Rosenfield AT, Riely CA. Ultrasonography of chronic liver disease with histological correlation. Radiology 1981;141:51-161
58. Chan CC, Hwang SJ, Lee FY, Wang SS, et al. Prognostic value of plasma endotoxin levels in patients with cirrhosis. *Scand J Gastroenterol* 1997;32:942-6
59. Fattovich G, Giustina G, Degos F, Tremolada, et al. Morbidity and mortality in compensated cirrhosis type C: A retrospective follow-up study of 384 patients. *Gastroenterology* 1997;112:463-72
60. Meek DR, Mills PR, Gray HW, Duncan JG, et al. A comparison of computerized tomography, ultrasound and scintigraphy in the diagnosis of alcoholic liver disease. *Br J Radiol* 1984;57:23-27
61. Biersack HJ, Reichmann K, Reske SN, Knopp R, et al. Improvement of scintigraphic liver imaging by SPECT- a review of 797 cases. *J Nucl Med* 1983;4: 282-9
62. Grime JS, Critchley M, Roberts M, Morris AI. Single photon emission tomography (SPECT) in the evaluation of chronic liver disease. *Nucl Med Cummun* 1983;4:282-9
63. Alstead EM, Grime JS, Critchley M, Morris AI, et al. Is SPECT of the spleen worthwhile in the evaluation of liver disease severity? *Nucl Med Commun* 1987:8:33-43
64. Shiomi S, Kurori T, Enomoto M, Ueda T, et al. Fulminanant hepatic failure monitored by technitium-99m-DTPA-galactosyl-human serum albumin scintigraphy. *J Nuc Med* 1996;37:641-3
65. Wu J, Ishikawa N, Takeda T, Tanaka Y, et al. The functional hepatic volume assessed by 99m Tc-GSA hepatic scintigraphy. *Ann Nuc Med* 1995;9:229-35
66. Hoefs JC. Wang F, Kanel G, et al. The liver-spleen scan as a quantitative liver function test: Correlation with liver severity at peritoneoscopy. *Hepatology* 1995;22:1113-21
67. Fusamoto H, et al. Progression of type C chronic hepatitis to liver cirrhosis and hepatocellular carcinoma- its relationship to alcohol drinking and the age of transfusion. *Alcohol Exp Res* 1996;20:92-100A
68. Merican I, Sherlock S, McIntyre N, Dusheiko GM. Clinical, biochemical and histological features in 102 patients with chronic hepatitis C virus infection. *Q J Med* 1993; 86:119-25
69. Perasso A, Testino G, Ansaldi F, Venturino V, et al.: r-Interferon alfa-2b/ribavirin combined therapy followed by low-dose r-interferon alfa-2b in chronic hepatitis C interferon nonresponders. *Hepatology* 1999;29:297-8
70. Gerber MA: Histopathology of HCV infection. *Clin Liver Dis* 1997; 1: 530-602
71. Gretch D, Corey I, Wilson J, et al: Assessment of hepatitis C virus RNA levels by quantitative competitive RNA polymerase chain reaction: high-titer viremia correlates with advanced stage of disease*. J Infect Dis* 1994;196:1219-25
72. Kato N, Yokosuka O, Hosoda K, et al: Quantification of hepatitis C virus by competitive reverse transcription polymerase chain reaction: Increase of the virus in advanced liver disease. *Hepatology* 1993;18:16-20
73. Dusheiko G, Weiss HS, Brown D, et al: Hepatitis C virus genotypes: An investigation of type-specific differences in geographic origin and disease. *Hepatology* 1994;19:13-18
74. Ichimura H, Tamura I, Kurimura O, et al: Hepatitis C virus genotypes, reactivity to recombinant immunoblot assay 2 antigens and liver disease. *J Med Virol* 1994;43:212-5

75. Farci P, Melpolder JC, Shimoda A, et al: Studies of HCV quasispecies in patients with acute resolving hepatitis compared to those who progress to chronic hepatitis. *Hepatology* 1996;24:350A
76. Honda M, Kaneko S, Sakai A, et al. Degree of diversity of hepatitis C quasispecies and progression of liver disease. *Hepatology* 1994;20:1144-1151
77. Alter HJ, Nakatsuji Y, Melpolder J, et al: The incidence of transfusion-associated hepatitis G virus infection and its relation to liver disease. *N Engl J Med* 1997;336:747-54
78. Alter MJ, Gallagher M, Morris TT, et al. Acute non-A-E hepatitis in the United States and the role of hepatitis G virus infection. *N Engl J Med* 1997;336:741-6
79. Pawlotsky JM, Roudot-Thoraval F, Pellerin M, et al. GBV-C infection in HCV-infected patients: Epidemiological characteristics, influence on HCV infection, and response to interferon alfa therapy. *Hepatology* 1996;24:226A
80. Tanaka E, Alter HJ, Nakatsuji Y, et al. Effect of hepatitis G virus infection on chronic hepatitis C. *Ann Intern Med* 1996;125:740-3
81. Tzonou A, Trichopoulos D, Kaklamani E, et al. Epidemiologic assessment of interactions of hepatitis C virus with seromarkers of hepatitis-B and -D viruses, cirrhosis, and tobacco smoking in hepatocellular carcinoma. *Int J Cancer* 1991; 49:377-80
82. Nakatsuji Y, Shih JWK, Kobayashi M, et al. Hepatitis G virus (HGV) infection in Japanese patients with hepatitis C virus (HCV). Hepatology 24:227A, 1996
83. Chiba T, Matsuzaki Y, Abei M, et al. The role of previous hepatitis B virus infection and heavy smoking in hepatitis C virus-related hepatocellular carcinoma. *Am J Gastroenterol* 1996;91:119-203
84. Fong TL, Di Bisceglie AM, Waggoner JG, et al. The significance of antibody to hepatitis C virus in patients with chronic hepatitis B. *Hepatology* 1991;14:64-7
85. Benvegnu L, Fattovich G, Noventa F, et al. Concurrent hepatitis B and C virus infection and risk of hepatocellular carcinoma in cirrhosis: a prospective study. *Cancer* 1994;27:2442-8
86. Coelho-Little ME, Jeffers LJ, Bernstein DE, et al. Hepatitis C virus in alcoholic patients with and without clinically apparent liver disease. *Alcohol Clin Exp Res* 1995;19:1173-6
87. Mendenhall CL, Seeff LB, Diehl AM, et al. Antibodies to hepatitis B virus and hepatitis C virus in alcoholic hepatitis and cirrhosis: Their prevalence and clinical relevance. *Hepatology* 1991;14:581-9
88. Oshita M, Hayashi N, Kasahara A, et al. Increased serum hepatitis C virus RNA levels among alcoholic patients with chronic hepatitis C. *Hepatology* 1994;20:1115-20
89. Sawada M, Takada A, Takase S, et al. Effects of alcohol on the replication of hepatitis C virus. *Alcohol* 1993;1B:85S-90S
90. Befrits R, Hedman M, Blomquist L, et al. Chronic hepatitis C in alcoholic patients: Prevalence, genotypes, and correlation to liver disease. *Scand J Gastroenterol* 30:1113-8
91. Jenkins PJ, Cromie SL, Roberts SK, et al. Chronic hepatitis C, alcohol and hepatic fibrosis. *Hepatology* 1996;24:153A
92. Matsuda Y, Amuro Y, Higashino K, et al. Relation between markers for viral hepatitis and clinical features of Japanese patients with hepatocellular carcinoma: Possible role of alcohol in promoting carcinogenesis. *Hepatogastroenterology* 1995;42:151-4
93. Noda K, Yoshihara H, Suzuki K, et al. Progression of type C chronic hepatitis to liver cirrhosis and hepatocellular carcinoma--its relationship to alcohol, drinking, and the age of transfusion. *Alcohol Clin Exp Res* 1996;20:95A-100A
94. Rosman AS, Paronetto F, Galvin K, et al. Hepatitis C virus antibody in alcoholic patients: Association with the presence of portal and/or lobular hepatitis. *Arch Intern Med* 1993;153:965-9
95. Hoofnagle JH, Di Bisceglie AM. The treatment of chronic viral hepatitis. *N Engl J Med* 1997;336:347-56
96. Poynard T, Bedossa P, Chevallier M, et al. A comparison of three interferon alfa-2b regimens for the long-term treatment of chronic non-A, non-B hepatitis. *N Engl J Med* 1995;332:1457-62

97. Carithers RL Jr, Emerson SS. Therapy of hepatitis C: meta-analysis of interferon alfa-2b trials. *Hepatology* 1997;26:83S-88S
98. Pestka SS. Langer JA, Zoon KC, Samual KC. Interferons and their actions. *Annu Rev Biochem* 1987;56:727-77
99. Gewert D, Flinter NB. Antiviral effects of interferons: studies in animal and at the cellular level. In: Baron S, Coppenhaver DH, Dianzani F, Fleischmann WR Jr, Hughes Tk Jr, Klimpel GR, Niesel DW, Santon GJ, Tyring SK, eds. Interferon Principles and Medical Applications. Galveston: University of Texas, 1992:289-297
100. Keeffe EB, Hollinger B, and the Consensus Interferon Study Group. Therapy of hepatitis C: consensus interferon trials. *Hepatology* 1997; 26:101S-107S
101. Lee WM. Therapy of hepatitis C: interferon alfa-2a trials. *Hepatology* 1997; 26: 89S-95S
102. Farrel GC. Therapy of hepatitis C: interferon alfa trials. *Hepatology* 1997; 26: 96S-100S
103. National Institute of Health Consensus Development Conference Panel Statement: Management of hepatitis C. *Hepatology* 1997; 26: 2S-10S
104. Lee WM, Reddy KR, Tong M, Black M, et al. Early hepatitis C virus RNA responses predict interferon treatment outcomes in chronic hepatitis C. *Hepatology* 1998; 28:1411-1415
105. Neary MP, Cort S, Bayliss MS, Ware JE Jr. Sustained virologic response is associated with improved health-related quality of life in relapsed chronic hepatitis C patients. *Semin Liver Dis* 1999; 19: 77S-85S
106. McHutchison JG, Gordon SC, Schiff ER, and the International Hepatitis Interventional Therapy Group: Interferon alfa 2b alone or in combination with ribavirin as initial treatment for chronic hepatitis C. *N Engl J Med* 1998;339:1485-92
107. Sostegni R, et al. Sequential versus concomitant administration of ribavirin and interferon alfa-n3 in patients with chronic hepatitis C not responding to interferon alone: results of a randomized, controlled trial. *Hepatology* 1998;28:341-6
108. Bellobuono A, et al. Ribavirin and interferon-alfa combination therapy vs interferon-alfa alone in the retreatment of chronic hepatitis C: a randomized clinical trial. *J Viral Hepat* 1997;4:185-91
109. Lai MY, Kao JH, Yang PM, et al. Long-term efficacy of ribavirin plus interferon alfa in the treatment of chronic hepatitis C. *Gastroenterology* 1996;111:1307-12
110. Shiffman ML, Hoffman CM, Thompson EB, et al. Relationship between biochemical, virological, and histological response during interferon treatment of chronic hepatitis C. *Hepatology* 1997;26:780-5
111. Bodenheimer HC, Lindsay KL, Davis GL, et al. Tolerance and efficacy of oral ribavirin treatment of chronic hepatitis C: a multicenter trial. *Hepatology* 1997;26:473-7
112. Alberti A, Chenello L, Noverta F, Cavalletto L, et al. Retreatment with interferon; proceedings of NIH Consensus Development Conference on the Management of Hepatitis C. *Hepatology* 1997;26:1375-1425
113. Tong MJ, Reddy RK, Lee WM, Pockros PJ, et al. Treatment of chronic hepatitis C with consensus interferon-a multicenter, randomized, controlled trial. *Hepatology* 1997;26:747-54
114. Heathcote EJ, Keefe EB, Lee SS, and the Consensus Interferon Study Group. Retreatment of chronic hepatitis C with consensus interferon. *Hepatology* 1998;27:1136-43
115. Heathcote J. Consensus interferon: a novel interferon for the treatment of hepatitis C. *J Viral Hepat* 1998;1:13-8
116. Lee WM, et al. Early hepatitis C virus-RNA responses predict interferon treatment outcomes in chronic hepatitis C. The Consensus Interferon Study Group. *Hepatology* 1998;28:1411-5
117. Bekkering FC, Brouwer JT, et al. Ultrarapid hepatitis C virus clearance by daily high-dose interferon in non-responders to standard therapy. *J Hepatol*. 1998; 28:960-4
118. Brass CA. Efficacy of interferon monotherapy in the treatment of relapsers and nonresponders with chronic hepatitis C infection. *Clin Ther* 1998;20:388-97

119. Rolachon A, et al. Value of high-dose interferon-alfa in chronic viral hepatitis C patients non-responder to a 1st treatment: pilot study prospective and randomized trial. *Gastroenterol Clin Biol* 1997;21:924-8
120. Chemello L, et al. Efficacy of a second cycle of interferon therapy in patients with chronic hepatitis C. *Gastroenterology* 1997;113:1654-9
121. Picciotto A. Long-term interferon alfa-2b retreatment of relapsing patients with chronic hepatitis C. *J Hepatol* 1997;26:447-8
122. Rabinovitz M, Block G, Finkelstein SD. Alpha interferon retreatment of patients with chronic hepatitis C. *Am J Gastroenterol* 1996;91:1523-6
123. Van Thiel DH, Friedlander L, et al. Retreatment of hepatitis C interferon non-responders with larger doses of interferon with and without phlebotomy. *Hepatogastroenterology* 1996; 43:1557-61
124. Marcellin P, Boyer N, Pouteau M, et al. Retreatment with interferon alpha of chronic hepatitis C virus infection. *Lancet* 1994; 344:690-1
125. Poynard T, Bedosa P, Chevallier M, et al. A comparison of three interferon alfa 2b regimens for the log-term treatment of chronic non-A; non-B hepatitis. *N Engl J Med* 1995;332:1458-62
126. Toyoda H, Nakano S, Takeda I, et al. Retreatment of chronic hepatitis C with interferon. *Am J Gastroenterol* 1994;89:1453-7
127. Lau DTY, Kleiner DE, Ghany MG, et al. 10 years follow-up after interferon alfa therapy for chronic hepatitis C. *Hepatology* 1998;28:1121-7
128. Jarmay K, et al. Histological evaluation of the response to interferon-alpha therapy in chronic hepatitis C. *Scand J Gastroenterol* 1998;228:56S-61S
129. Kumagai N, et al. Correlation between histological features of liver biopsy specimens and clinical effect of interferon on patients with chronic hepatitis C. *Nippon Rinsho* 1994;52:1769-73
130. Bonis PA, et al. Correlation of biochemical response to interferon alfa with histological improvement in hepatitis C: a meta-analysis of diagnostic test characteristics. *Hepatology* 1997;26:1035-44
131. Shindo M, et al. The clinical value of grading and staging scores for predicting a long-term response and evaluating the efficacy of interferon therapy in chronic hepatitis C. *J Hepatol* 1997;26:492-7
132. Panella C, et al. Clinical and histological evaluation of interferon alfa-2b in the treatment of chronic hepatitis C. *Gut* 1993;34:S127
133. Kumagai N, et al. Assessment of histological features and outcome of interferon therapy in chronic hepatitis C. *J Gastroenterol* 1996;31:69-74
134. Tsubota A, et al. Time course of histological changes in patients with a sustained biochemical and virological response to interferon-alpha therapy for chronic hepatitis C virus infection. *J Hepatol* 1997;27:49-55
135. Yokosuka O, et al. Histological assessment of the liver long after interferon treatment in patients with chronic type C hepatitis. *Nippon Rinsho* 1994; 52:1841-6
136. Marcellin P, et al. Long-term histologic improvement and loss of detectable intrahepatic HCV RNA in patients with chronic hepatitis C and sustained response to interferon-alpha therapy. *Ann Intern Med* 1997;127:875-81
137. Ninomiya T, et al. Clinical significance of serum hyaluronic acid as a fibrosis marker in chronic hepatitis C patients treated with interferon-alpha: histological evaluation by a modified histological activity index scoring system. *J Gastroenterol Hepatol* 1998;13:68-74
138. Dufour JF, et al. Regression of hepatic fibrosis in hepatitis C with long-term interferon treatment. *Dig Dis Sci* 1998;43:2573-6
139. Kasahara A, Hayashi N, et al. Risk factors for hepatocellular carcinoma and its incidence after interferon treatment in patients with chronic hepatitis C. *Hepatology* 1998;27:1394-1402
140. Onodera H, et al. Incidence of hepatocellular carcinoma after interferon therapy in patients with chronic hepatitis C. *Tohoku J Exp Med* 1997;18:275-83

141. Imai Y, et al. Relation of interferon therapy and hepatocellular carcinoma in patients with chronic hepatitis C. *Ann Intern Med* 1998;129:94-9
142. Kuwana K, et al. Risk factors and the effect of interferon therapy in the development of hepatocellular carcinoma: a multivariate analysis in 343 patients. *J Gastroenterol Hepatol* 1997;12:149-55
143. Saracco G, Borgheshio E, Mesina P, Solinas A, et al. Prolonged treatment (2 years) with different doses (3 versus 6 MU) of interferon alfa-2b for chronic hepatitis type C. Results of a multicenter randomized trial *J Hepatol* 1997;27:56-62
144. Sanchez-Tapias JM, Forns X, Ampurdanes S, et al. Low dose alpha interferon therapy can be effective in chronic active hepatitis C. Results of a multicentre, randomised trial. *Gut* 1996;38:603-9
145. Davis GL, Balart LA, Schiff ER, Lindsay K, et al. Treatment of chronic hepatitis C with recombinant interferon alpha: a multicenter, randomized, controlled trial. N Eng J Med 1989;321:1501-1506
146. Di Bisceglie AM, Martin P, Kassianides C, Lisker-Melman M, et al. Recombinant interferon alfa therapy for chronic hepatitis C. N Eng J Med 1989; 321:1506-1510
147. Marcellin P, Boyer N, Giostra E, Degott C, et al. Recombinant human alpha-interferon in patients with chronic non-A, non-B hepatitis: A multicenter randomized controlled trial from France. *Hepatology* 1991; 13:393-397
148. Causse X, Godinot H, Chevallier M, Chossegros P, et al. Comparison of 1 or 3 mU of interferon alpha-2b and placebo in patients with chronic non-A, non-B hepatitis. *Gastroenterology* 1991; 101:497-502
149. Chemello L, Bonetti P, Cavalletto L, Talato F, et al. Randomized trial comparing three different regimens of alfa-2a-interferon in chronic hepatitis C. *Hepatology* 1995; 22:700-706
150. Poynard T, Bedossa P, Chevallier M, Mathurin P, et al. A comparison of three interferon alfa-2b regimens for the long-term treatment of chronic non-A, non-B hepatitis. *N Eng J Med* 1995; 332:1457-1462
151. Shiffman ML, Hofmann CH, Luketic, VA, Sanyal AJ, et al. Gradual reduction in interferon dose reduces the incidence of relapse in patients with chronic hepatitis C: Results of a randomized controlled trial. *Hepatology* 1996; 24:21-26
152. Shiffman ML, Hofmann CM, Thompson EB, Ferreira-Gonzalez A, et al. Relationship between biochemical, virologic and histologic response during interferon treatment of chronic hepatitis C. *Hepatology* 1997; 26:780-785
153. Ishak KG. Hepatic histopathology. In *Diseases of the Liver*. Schiff L and Schiff ER (eds). Lippincott Co, Philadelphia, 1993, pp. 145-188
154. Boyer JL, Reuben A. Chronic hepatitis. In *Diseases of the Liver*. Schiff L and Schiff ER (eds). Lippincott Co, Philadelphia, 1993, pp. 586-637
155. Gerber MA. Histopathology of HCV infection. *Clin Liv Dis* 1997; 1:529-541
156. Dietrichson O, Christoffersen P. The prognosis of chronic aggressive hepatitis. *Scand J Gastroenterol* 1977; 12:289-295
157. Yano M, Kumada H, Kage M, Ikeda K, et al. The long term pathological evolution of chronic hepatitis C. *Hepatology* 1996; 23:1334-1340
158. Shiffman ML, Hofmann CM, Sanyal AJ, Luketic VA, et al. The effect of maintenance interferon in chronic HCV non-responders: Results of a randomized controlled trial. *Gastroenterology* 1999; (in press)
159. Nishiguchi S, Kuroki T, Nakatani S, et al. Randomised trial of effects of interferon on incidence of hepatocellular carcinoma in chronic active hepatitis C with cirrhosis. *Lancet* 1995;346:1051-1055.
160. Schalm SW, Fattovich G, Brouwer JT. Therapy of hepatitis C: Patients with cirrhosis. *Hepatology* 1997;26 (Suppl 1):128S-132S.
161. Fattovich G, Giustina G, Degos F, et al. Effectiveness of interferon alfa on incidence of hepatocellular carcinoma and decompensation in cirrhosis type C. *J Hepatol* 1997;27:201-205.

162. Mazzella G, Accogli E, Sottili S, et al. Alpha interferon treatment may prevent hepatocellular carcinoma in HCV-related liver cirrhosis. *J Hepatol* 1996;24:141-147
163. Yoshida H, Shiratori Y, Moriyama M, et al. Interferon therapy reduces the risk for hepatocellular carcinoma: National surveillance program of cirrhotic and noncirrhotic patients with chronic hepatitis C in Japan. *Annals of Internal Medicine*. 1999;131:174-181
164. Bruno S, Silini E, Crosignani A, et al. Hepatitis C virus genotypes and risk of hepatocellular carcinoma in cirrhosis: A prospective study. *Hepatology* 1997;25:754-758
165. Silini E, Bottelli A, Asti M, et al. Hepatitis C virus genotypes and risk of hepatocellular carcinoma in cirrhosis: a case-control study. *Gastroenterology* 1996;111:199-205
166. Fattovich G, Giustina G, Degos F, et al. Morbidity and mortality in compensated cirrhosis type C: A retrospective follow-up study of 384 patients. *Gastroenterology* 1997; 112:463-472
167. Serfaty L, Aumaitre H, Chazouilleres O, et al. Determinants of outcome of compensated hepatitis C virus-related cirrhosis. *Hepatology* 1998;27:1435-1440
168. Shiffman M, Pockros PJ, Reddy RK, et al. A controlled, randomized, multicenter, descending dose phase II trial of pegylated interferon alfa-2A (PEG) vs standard interferon alfa-2A (IFN) for treatment of chronic hepatitis C (abstract). *Gastroenterology* 1999;116:A1275
169. McHutchison JG, Gordon SC, Schiff ER, et al. Interferon alfa-2b alone or in combination with ribavirin as initial treatment for chronic hepatitis C. *New England Journal of Medicine* 1998;339:1485-1492
170. Poynard T, Marcellin P, Lee SS, et al. Randomized trial of interferon alfa-2b plus ribavirin for 48 weeks or for C virus. *Lancet* 1998;352:1426-1432
171. Friedman LM, Furberg CD, DeMets DL. Fundamentals of Clinical Trials, 3rd ed., Mosby-Year Book , Inc. St. Louis, MO, 1996
172. Lehmann EL. Nonparametrics: statistical methods based on ranks. New York, NY: McGraw-Hill 1975
173. Agresti A. Analysis of categorical data. New York: John Wiley 1990
174. Fleiss JL. Statistical methods for rates and proportions. New York: John Wiley 1981
175. Finkelstein DM. A proportional hazards model for interval-censored failure time data. Biometrics 1986; 42:845-854
176. Lindsay JC and Ryan LM. Tutorial in biostatistics: methods for interval-censored data. Statistics in Medicine 1998; 17:219-238
177. Sun J. Regression analysis of interval-censored failure time data. *Statistics in Medicine* 1997; 16:497-504
178. Turnbull BW. The empirical distribution function with arbitrarily grouped, censored and truncated data. Journal of the Royal Statistical Society, Series B 1976; 38:290-295
179. O'Brien PC and Fleming TR. A multiple testing procedure for clinical trials. Biometrics, 1979; 35:5490566
180. Lan KKG and DeMets DL. Discrete sequential boundaries for clinical trials. Biometrika 1983; 70:659-663
181. DeMets L and Lan KKG. Interim analysis: the alpha spending function approach. Statistics in Medicine 1994; 13:1341-1352
182. Diggle PJ, Kiang KY, and Zeger SL. Analysis of Longitudinal Data. Oxford University Press, New York, NY, 1994

APPENDICES

A. Definitions

Ascites: Any abdominal fluid which is:

1. Mild, moderate or marked on ultrasound; or
2. Progressive on serial physical examinations; or
3. Requires diuretic therapy.

To meet the definition of ascites, abdominal fluid that is "mild" ("barely detectable") on physical examination requires ultrasound confirmation that is "mild", "moderate" or "marked" ascites. Ultrasound reports of minimal fluid around the liver do not meet the definition.

Hepatic encephalopathy: Any mental status alteration which is deemed by the investigator to be due to portosystemic encephalopathy, whether occurring during a provoked episode (GI bleeding, diuretics, usual sedative doses), or spontaneously (without apparent cause).

Hepatocellular carcinoma: A diagnosis of HCC will be based on either

1. Histology showing HCC (from a biopsy, surgery, or autopsy) or
2. A new hepatic defect on imaging with an AFP rising to > 1,000 ng/ml.

Hepatorenal syndrome: Progressive deterioration in renal function, with no other etiology, rising serum creatinine to > 1.5 mg/dl and at least one of the following;

1. Urine volume < 500 ml/d
2. Urine sodium < 10 mEq/l
3. Urine osmolality > plasma osmolality > 1

Presumed Hepatocellular Carcinoma: Presumed HCC will be considered when histology is not available and AFP is <1000 ng/ml, if:

1. A new hepatic lesion is shown on ultrasound and one additional imaging which shows a hepatic lesion with characteristics of HCC.
2. AFP >ULN and two imaging studies show a hepatic lesion with characteristics of HCC.
3. A progressively enlarging hepatic lesion starting as a new defect eventually resulting in the death of a patient.
4. A new hepatic defect with at least 1 characteristic scan and one of the following:
    a. Increase in size over time (doubling in diameter or tripling in diameter if the initial size <1 cm when first discovered) or
    b. An increasing AFP (values 3 months before or after the discovery of the defect by scanning) eventually rising to a level of >200 ng/ml and more than tripling the mean baseline value.

Images may include:

1. MRI
2. triphasic CT
3. angiography (angiography taken prior to intra-arterial chemo-embolization may be used for this purpose if images are taken and reported prior to therapeutic intervention)
4. lipiodol scan
5. liver spleen scan with gallium

Characteristics of HCC include:

1. Hypervascularity
2. Arterial to portal vein shunts
3. Portal vein thrombosis near the defect
4. Tumor in the portal vein.

Spontaneous bacterial peritonitis: Any episode of spontaneous ascitic infection diagnosed on the basis of elevated neutrophil count (> 250/ml) in paracentesis fluid or positive bacterial cultures and clinical diagnosis in the absence of WBC availability.

Variceal hemorrhage: A gastrointestinal hemorrhage which is believed by the investigator to be due to bleeding esophageal or gastric varices. In general, an endoscopy will have been performed and will have revealed either direct evidence of variceal bleeding (bleeding varix, red wale sign) or historical evidence for significant upper gastro-intestinal bleeding plus upper endoscopy revealing moderate varices and no other site of bleeding is identified.

UNOS transplant listing as Status 2b (as of 1999):

1. Presence of a small hepatocellular carcinoma; or
2. CTP score of 10 or more; or
3. CTP score of 7 or more plus any one of the following:
    - Documented unresponsive variceal hemorrhage
    - Hepatorenal syndrome
    - Occurrence of one episode of SBP
    - Refractory ascites or hepato-hydrothorax unresponsive to treatment

B. Child-Turcotte-Pugh Score for Grading Severity of Liver Disease

Modified Child-Turcotte-Pugh Score				
		# of points		
**Variable**	**Units**	**1**	**2**	**3**
Serum albumin	(g/dL)	>3.5	2.8-3.5	<2.8
Serum total bilirubin<br>(No Gilbert's Syndrome;<br>No hemolytic diseases;<br>Not receiving ribavirin)	(mg/dL)	<2.0	2.0-3.0	>3.0
Serum total bilirubin<br>(In presence of Gilbert's Syndrome, a hemolytic disorder [e.g., patients receiving ribavirin]) [‡]	(mg/dL)	<4.0	4.0-7.0	>7.0
Prothrombin Time	(INR)	<1.7	1.7-2.3	>2.3
Ascites		None	mild*	severe+
Encephalopathy		None	mild*	severe+

*Mild means readily controlled by standard medical therapies.

+Severe means difficult to control or uncontrollable by optimal, maximally tolerated medical therapies.

Prothrombin time results should be reported and used for calculations only as International Normalized Ratios (INR), because of variations in methods used and reference ranges for controls (expressed in seconds).

‡ Note that if, in the opinion of the investigator, the patient has Gilbert's syndrome or a hemolytic disorder (e.g., patients receiving ribavirin) the level of the serum total bilirubin may be increased to as high as 3.99 mg/dL without considering the total bilirubin to be sufficiently elevated for the patient to receive a score of 2 in the CTP scoring system.

The score is calculated as the sum of the scores for albumin, bilirubin, prothrombin time, ascites and encephalopathy (range 5-15). Class A is defined as 5-6, class B 7-9 and class C 10-15.

C. Management Guidelines

C.1. Depression

Background
Mood disorders (i.e., depression and anxiety) are common and problematic side effects of IFN. Mood disorders may develop during the first few months of treatment or later during long term therapy. Mood disorders can develop in individuals without a prior psychiatric history and present with subtle signs and symptoms such as fatigue, withdrawn behavior, poor appetite, irritability, or sleep disturbance. Although IFN dose reduction and anti-depressant medications have proven useful, severe and life-threatening neuropsychiatric toxicity including attempted and completed suicide have been reported (1-3). Therefore, a heightened awareness of depression and its potential impact on patient safety and compliance is necessary when prescribing interferon.

Detection of Depression
In the HALT-C Trial, the CIDI will be administered pretreatment and the Beck Depression Index-II (BDI-II) will be administered every 3 months during treatment. In order to provide rapid and reliable psychiatric services for enrolled patients, a collaborative psychiatrist/ psychologist should be identified in each center for urgent and elective referrals.

CIDI
The CIDI-Auto 2.1 is the computerized version of the Composite International Diagnostic Interview (CIDI) developed by the World Health Organization (4). The CIDI is a comprehensive, fully standardized interview that can be used to assess mental disorders and provide diagnoses according to the definitions and criteria of the ICD-10 and DSM-IV (See Attachment 1). The CIDI can be administered by trained study personnel, does not require outside informants or medical records, and can be completed in 20-40 minutes.

Beck Depression Index-II (BDI-II)
The BDI-II is a 21 item, self-administered survey used to screen for and monitor depression that takes 5-10 minutes to complete (5). The BDI-II has been shown to provide valid and reliable information in follow-up studies of patients with either psychiatric illness or medical illness (6,7). Although no arbitrary scores are available that can be used on all patients to classify the severity of depression, specific interpretation guidelines are available (8). The BDI-II was developed for the assessment of symptoms corresponding to DSM-IV criteria for diagnosing depressive disorders in 1996. The BDI-II has been extensively tested and validated and is felt to be an improvement over the previous versions of the Beck(9).

Screening

1. Subjects with the following psychiatric disorders will be excluded from the trial:
   - Suicide attempt or hospitalization for depression within the past 5 years
   - Any current (within 6 months) severe or poorly controlled psychiatric disorder (e.g., depression, schizophrenia, bipolar illness, obsessive-compulsive disorder, severe anxiety, personality disorder).
2. The following patients must be assessed and followed (if recommended) by a psychiatrist or other mental health professional:
   - Patients who have had a suicide attempt and/or hospitalization for depression more than 5 years ago,
   - Patients who have had severe or poorly controlled psychiatric disorder (e.g., depression, schizophrenia, bipolar illness, obsessive-compulsive disorder, severe anxiety, personality disorder) more than 6 months ago but less that 5 years ago.

   Those patients unwilling to be assessed and followed in this manner will not be eligible for the trial.

3. At screen 1, eligible subjects with a history of severe or dose limiting-neuropsychiatric toxicity during prior interferon treatment should be referred to the collaborative psychiatrist/ psychologist. In addition to clarifying possible psychiatric disorders, the consultant may advise on the need for adjuvant medical or counseling therapy and suitability for enrollment in the HALT-C Trial.

4. During screening, all eligible subjects will complete the Anxiety, Depression, Alcohol, and Substance abuse modules of the CIDI auto 2.1, except for those site with special difficulties (see G.3.c.)

5. Subjects with a DSM-IV diagnosis of recent panic disorder, recent generalized anxiety disorder, and recent major depression from the CIDI should be evaluated by the Principal Investigator or physician co-investigator (See Attachment 1, page 58).

6. At screen 2 all eligible subjects will complete the BDI-II (see below).

Monitoring of Depression

1. Potential mood disturbance/medication intolerance will be assessed at each visit by study personnel.

2. Anemia, thyroid dysfunction, and other confounding medical issues should be evaluated in any patient with a mood disorder or abnormal BDI-II score.

3. The BDI-II will be self-administered at Screening and every 3 months to month 54. It will be scored and interpreted locally at each study visit and the score will be recorded in the HALT- C database (Form #44).

4. The BDI-II is scored by summing the ratings for 21 items. Each item is rated on a 4 point scale ranging from 0-3.

5. Subjects with abnormal BDI-II scores (range: 11 to 63 with higher scores indicative of more severe symptoms) should be assessed and managed as follows:

BDI-II score	Clinical Picture
0 – 10	none to minimal
11 - 14	Mild depression
15 - 19	Mod depression
20 - 28	Severe depression
≥29	Critical

6. Practitioners should keep in mind that all self-report inventories are subject to response bias. That is, some individuals may endorse more symptoms then they actually have and thus produce spuriously high scores while others might deny symptoms and receive spuriously low scores. In addition, the practitioner is cautioned that the BDI-II may simply reflect the degree of depression, not the diagnosis of depression. Determination of the severity of depression and the establishment of a diagnosis of depression require examination by a clinician (physician or psychologist/ psychiatrist).

7. Because the BDI-II total score provides only an estimate of the overall severity of depression, it is important to be attentive to specific items regarding suicidal ideation. Patients admitting to suicide ideation **(Item 9**) and hopelessness **(Item 2)** with a **rating of 2 or 3** should be closely scrutinized for suicide potential.

8. Any patient who develops recurrent suicidal ideation, a suicide plan and/ or makes a suicide attempt should have IFN immediately discontinued and be referred to a psychiatrist for further management.

9. Suggested guidelines for interferon dose reduction:
   - Unpleasant and/or disabling side effects might prompt dose reduction, but this is not required.
   - A BDI score of 20-28 or a score of 15-19 that has doubled since the previous measurement might prompt further attention from the PI, but is not a requirement for dose reduction.
   - A score of >29 or higher should prompt attention from the PI and dose reduction or discontinuation should be considered.

10. Suggested guideline for withholding/discontinuing interferon:
    - Persistent suicide ideation/suicide plan/suicide attempt should lead to discontinuation of interferon.

11. Follow-up telephone contact is recommended for all patients within 2 to 4 weeks if :
    - BDI-II > 15
    - anti-depressives are started
    - interferon is reduced due to psychiatric toxicity

Anti-depressants

1. For subjects with moderate or severe depressive symptoms or with a BDI-II score of > 15, anti-depressives should be used before considering interferon dose reduction, whenever possible.

2. Subjects requiring anti-depressants will likely require additional telephone follow-up and clinic visits for depression assessment per local PI.

3. Anti-depressant medication selection will be determined by patient tolerance and physician preference.

4. The following table provides information on 2 available anti-depressants which may be of use in treating interferon induced depressive symptoms:

**Drug**	**Citalopram** (Celexa, P-D)	**Venlafaxine XR** (Effexor XR, Wyeth)
**Starting dose**	20 mg qd	37.5 mg qd (50% in cirrhosis)
**Maximum dose**	60 mg qd	150 mg qd
**Mechanism of action**	Selective SSRI	SSRI and norepi reuptake inhibitor
**Side effects**	Dry mouth, nausea, sleep disturb	Anxiety, sweating, nausea
**Advantages**	Once a day	Once a day
**Precautions**	CYP3A4 drug interactions: avoid MAO inhibitors	CYP2D6 drug interactions: avoid MAO inhibitors
**Drug cost 1 mon**	$78	$75

Psychiatric referral
Referral to the identified local psychiatry collaborator should be considered if patients develop mood disturbance refractory to anti-depressants or other interventions and/or suicidal thoughts or intent.

References

1. McHutchison JG et al. Interferon-a2b alone or in combination with ribavirin as initial treatment for chronic hepatitis C. *NEJM* 1998; 339: 1485-1492
2. Dusheiko G. Side effects of alpha interferon in chronic hepatitis C. *Hepatology* 1997; 26(Suppl): 112S-121S
3. Renault PF, et al. Psychiatric complications of long-term interferon therapy. *Arch Intern Med* 1987; 147; 1577-1580
4. Wittchen HU. Reliability and validity studies of the WHO Composite International Diagnostic Interview: A critical review *J Psychiatric Research* 1994; 28: 57-84
5. Beck A, Ward C, Mendelsohn M, et al. An inventory for measuring depression. *Arch Gen Psychiatry* 1961; 4: 561-571
6. Thase M, Simmons A, Cahalane J, et al. Severity of depression and response to cognitive behavior therapy. *Am J Psychiatry* 1991; 148: 784-789
7. Borras C, Rio J, Porcal J, et al. Emotional state of patients with relapsing-remitting multiple sclerosis treated with interferon beta 1-b. *Neurology* 1999; 52: 1636-9
8. Spreen D, Strauss E A compendium of neuropsychological tests, administration, norms, and commentary. New York, Oxford University Press, 1998; 601-8
9. Beck AT, Steer RA, Brown GK. BDI-II Manual. Harcout Brace & Company, San Antonio, TX, 1996

Attachment 1

DSM-IV Diagnosis from CIDI-Auto 2.1 Interview

Anxiety disorders

Specific phobia
Social phobia
Agoraphobia without history of panic disorder
* Panic disorder without and with agoraphobia
* Generalized anxiety disorder

Depressive disorders

* Major depression, single episode (subtypes: mild, moderate, severe)
* Major depression: recurrent (subtypes: mild, moderate, severe)
Dysthymia

Alcohol abuse

Alcohol dependence

Psychoactive substance use disorders; dependence or abuse

Cannabis	Opioid	Inhalant
Cocaine	Sedatives	PCP
Hallucinogen	Stimulants	Amphetamine or similar-acting substance

Others (not otherwise specified [NOS])

C.2. HCC Screening

Introduction

The purpose of obtaining the screening AFP and ultrasound (U/S) is to document that hepatocellular carcinoma (HCC) is not present during the screening period. A normal AFP and absence of a defect by U/S is strong evidence against the presence of HCC.

The higher the AFP, the more likely that undetected HCC is present. However, 20–30 % of patients with HCV uncomplicated by HCC will have elevations of AFP above the normal range. Most of these elevations are relatively low (< 75 ng/ml) and stable. Stable levels are defined as less than twice the initial value over 28 days or more. Furthermore, stable values between 75 and 200 ng/ml are often not due to HCC if the levels are not rising and the U/S is negative for a defect.

Testing Schedule

AFP testing is to be performed at screen 1, baseline, and every three months. Ultrasound is to be performed during screening (if not performed during the previous 6 months), at week 20, and then at months 12, 24, 36, and 48 for randomized patients.

AFP Monitoring during Screening

1. If the AFP is between ULN and 75 and the ultrasound is normal, then the patient is eligible for the Lead-in.
2. If the AFP is between 76 and 200, then the ultrasound must be normal and an MRI or CT must be normal for the patient to be eligible for the Lead-in.
3. If the AFP is > 200, then the patient is not eligible for the Lead-in.
4. If the Screening AFP for Express patients is between 200 and 1000, the ultrasound must be normal and an MRI or CT must be normal for the patient to be eligible for enrollment.
5. If for some reason the required tests cannot be performed within the time limit from Screening Visit 1 to baseline, then the site may submit a formal request to the Exemption Committee to extend the visit window.

AFP Monitoring during the Lead-in and Randomized Phases

The following abnormal findings should trigger concern about possible HCC and require additional workups:

1. A doubling of screening or baseline value or a value of >200.

The additional work-up in response to abnormal findings should include:

1. AFP testing
   Perform AFP testing monthly until there is a 50% decrease or it remains stable for 3 months, and
2. Ultrasound
   a. If the ultrasound was performed in the previous 3 months, perform triphasic CT or MRI.
   a. If the ultrasound was not performed within the last 3 months, repeat the ultrasound.
   c. If the repeat ultrasound shows new defect, perform triphasic CT or MRI.

d. If the repeat ultrasound shows no defect and AFP continues to rise
   - Perform triphasic CT or MRI
   - If triphasic scan or MRI are negative for HCC, perform monthly AFP until there is a >50% decline or it remains stable over 3 months, or if elevated, repeat MRI.

C.3. Portal Hypertension

Portal hypertension is a major complication of cirrhosis and contributes substantially to cirrhosis-related morbidity and mortality, which are important endpoints for the HALT-C Trial. It is therefore important that the management of portal hypertension in patients enrolled in this trial be standardized across treatment centers and treatment arms. Such management will be considered below in the context of the natural history of portal hypertension and variceal hemorrhage:

1. Management of patients who have never bled from varices before (Attachment 2):

The plan of management of such patients will depend on the histologic stage of their disease. Clinically significant portal hypertension does not occur in patients with hepatitis c until cirrhosis develops. Therefore all patients will undergo a diagnostic endoscopy within 4 weeks of randomization (unless one has been done within the last 12 months).

A. Initial endoscopy:

The objective of this initial baseline endoscopy will be to document the presence or absence of esophageal varices, gastric varices and portal gastropathy. This will provide valuable baseline data that will be used for comparison with findings from subsequent endoscopies.

1. Methods:

<u>Panendoscopy</u> will be performed using conscious sedation in most cases. Appropriate safety guidelines, as defined by the American Society of Gastrointestinal Endoscopy will be used during the procedure. The following will be assessed:

a. <u>Esophageal varices</u> will be assessed in the distal 5 cm of the esophagus with air-insufflation of the esophagus. The following parameters will be noted:
   1. # of columns of varices
   2. extent of varices
   3. size of varices: corresponding to F1-F3 of the NIEC classification, as below:
      <u>Grade 0 (none)</u>: No varices present.
      <u>Grade 1 (small)</u>: Varices that can be flattened out by insufflation.
      <u>Grade 2 (medium)</u>: Varices that can not be flattened out by insufflation and which occupy less that 33% of the lumen of the esophagus.
      <u>Grade 3 (large)</u>: Varices that occupy more than 33% of the lumen of the esophagus.
   4. Red signs: red wale marks, cherry red (hematocystic) spots, varix on varix

b. Gastric varices will be identified and classified according to Sarin's classification as:
   1. Gastro-esophageal varices (GOV) type 1: Gastric varices in continuity with esophageal varices along the lesser curve of the stomach.
   2. Gastro-esophageal varices (GOV) type II: Gastric varices in continuity with esophageal varices along the greater curve of the stomach.
   3. Isolated gastric varices (IGV) type I: Isolated cluster of varices in the fundus of the stomach
   4. Isolated gastric varices (IGV) type II: Isolated varices in regions of the stomach other than in the fundus.

c. Endoscopic assessment and scoring of portal gastropathy will be defined as follows for the purposes of this study (These definitions are identical to those proposed by the NIEC (1) and by Sarin (2) to develop the scoring system for portal gastropathy presented at the Baveno conference):
   1. Mosaic pattern: small polygonal areas demarcated by a distinct white-to-yellow border and with a slight central bulge, which have a mosaic, fish scale-like appearance upon endoscopy. The mosaic pattern will be considered to be mild when the color of the mucosa is pink while diffuse erythema (redness) of the mucosa will be considered to represent severe MP.
   2. Red Marks: flat or slightly bulging red lesions seen in the gastric mucosa. Such lesions include fine punctuate hemorrhagic spots and discrete red spots corresponding to the red point lesions and cherry red spots described by the NIEC group **(**1). When present in isolated discrete spots, they will be given a score of 1 while confluent areas of submucosal hemorrhage will be given a score of 2 (table 1).
   3. Black-brown spots: represent old submucosal hemorrhage and will not be scored.
   4. Gastric antral vascular ectasia (GAVE): will be diagnosed by the presence of flat or slightly raised red stripe-like lesions radiating from the pylorus to the antrum and body of the stomach for a variable distance (3).

2. Calculation of the portal gastropathy score

The severity of the portal gastropathy will be scored as proposed by Sarin (2). A mild mosaic pattern will be given a score of 1 while severe MP will be scored as 2. Isolated RM will be scored as 1 while confluent RM will be scored as 2. Absence of GAVE will be scored as 0 while the presence of GAVE will be scored as 2. The portal gastropathy will be considered to be mild when the total score is less than or equal to 3 and severe if the score is 4 or greater.

3. Photodocumentation of endoscopy

Photographs of each endoscopy will be taken. Two sets of photographs will be required. One set will remain on site in the patient's record. The second will be sent to the DCC for blinded review. The photographs will be reviewed in a blinded manner by a panel of endoscopists at a central location and scored separately by each member of the panel.

4. Management of patients following initial endoscopy:

As outlined in Attachment 2 (page 63), the management of patients will depend on the findings of the initial endoscopy. Those without varices will be followed without additional interventions. All those with Grade 2 (medium) or Grade 3 (large) esophageal varices as well as gastric varices will be treated for primary prophylaxis of variceal hemorrhage as follows:

> Agent to be used: nadolol or another non cardio selective beta blocker if nadolol cannot be used.
> Dosage: Treatment will be initiated with 20-40 mg/day orally (once a day) and the dose titrated upwards until the resting heart rate is between 55-65 beats/min or adverse effects occur preventing further increase or continuation of therapy.
> Subsequent endoscopy: will be performed at month 48 (exit) for those who have no varices at initial endoscopy. In those with varices, endoscopy will be performed again at months 24 and 48**.**These times were determined on the basis of the probability of finding differences in the two treatment arms at the different points in time from entry.
> What to do for those with large varices who are intolerant of beta blockers: It is anticipated that up to 15-20% of subjects who are started on beta blockers will have adverse effects requiring discontinuation of the drug. In such cases, endoscopic band ligation may be performed at the investigators' discretion and after discussion with the patient. Such an option will be necessary because of ethical concerns about withholding potentially life-saving therapy in such subjects.

5. Management of active variceal hemorrhage:

Management of active variceal hemorrhage or prevention of recurrent variceal hemorrhage should be performed according to the standard of care at each institution

BIBLIOGRAPHY

1. Spina, G. P., R. Arcidiacono, J. Bosch, L. Pagliaro, et al. 1994. Gastric endoscopic features in portal hypertension: final report of a consensus conference, Milan, Italy, Sept 19, 1992. *J Hepatol* 21:461-467
2. Sarin, S. K. 1996. Diagnostic issues: portal hypertensive gastropathy and gastric varices. In Portal hypertension II. Proceedings of the second Baveno international consensus workshop on definitions, methodology and therapeutic strategies. R. DeFranchis, editor. Blackwell Science, Oxford. 30-55
3. Gostout, C. J., T. R. Viggiano, D. A. Ahlquist, K. K. Wang, et al. 1992. The clinical and endoscopic spectrum of the watermelon stomach. *J Clin Gastroenterol* 15:256-263
4. Grace, N. D. 1997. Diagnosis and treatment of gastrointestinal bleeding secondary to portal hypertension. American College of Gastroenterology Practice Parameters Committee. *Am. J. Gastroenterol.* 92:1081-1091
5. D'Amico, G., A. Morabito, and L. Pagliaro. 1986. Six week prognostic indicators in upper gastrointestinal hemorrhage in cirrhotics. *Frontiers of Gastrointestinal Research* 9:247-258

Attachment 2

Management of patients who have never bled from varices

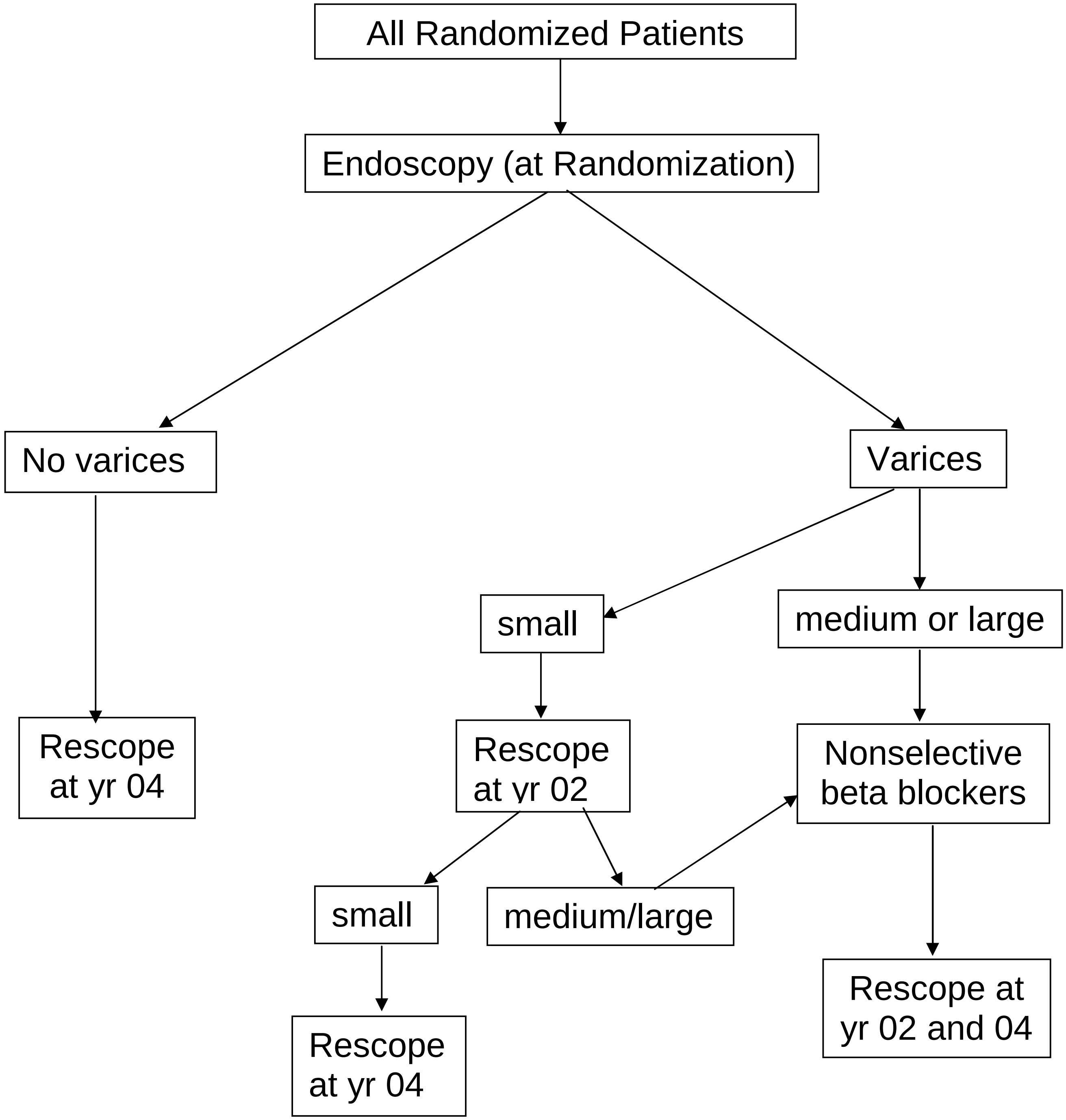

D. Adverse Events

D1. Relationship of adverse event to trial medication

Unrelated: This category is applicable to those adverse events which, after careful medical consideration at the time of evaluation, are judged to be clearly and incontrovertibly due to extraneous causes (disease, environment, etc.) and do not meet the criteria for drug relationship listed under Remote, Possible or Probable.

Remote: (must have a + b) In general, this category is applicable to an adverse event which meets the following criteria:

a. It does not follow a reasonable temporal sequence from administration of the Trial medication.
b. It could readily have been produced by the patient's clinical state, environmental or toxic factors, or other modes of therapy administered to the patient.
c. It does not follow a known pattern of response to the suspected drug.
d. It does not reappear or worsen when the drug is re-administered.

Possible: (must have a + b) This category applies to those adverse events in which the connection with Trial medication administration appears unlikely, but cannot be ruled out with certainty.

a. It follows a reasonable temporal sequence from administration of Trial medication.
b. It may have been produced by the patient's clinical state, environmental or toxic factors, or other modes of therapy administered to the patient.
c. It follows a known pattern of response to the suspected drug.

Probable: (must have a, b + c) This category applies to those adverse events that are considered, with a high degree of certainty, to be related to Trial medication (either interferon or ribavirin). An adverse event may be considered probable if:

a. It follows a reasonable temporal sequence from administration of the Trial medication.
b. It could not be reasonably explained by the known characteristics of the patient's clinical state, environmental or toxic factors, or other modes of therapy administered to the patient.
c. It disappears or decreases on cessation or reduction in dose. (There are important exceptions when an adverse event does not disappear upon discontinuation of the drug, yet drug relatedness clearly exists; e.g., 1) bone marrow depression; 2) tardive dyskinesias.)
d. It follows a known pattern of response to the suspected drug.
e. It reappears upon rechallenge.

	Unrelated	Remote	Possible	Probable
Clearly due to extraneous causes	+	-	-	-
Reasonable temporal association Trial drug administration	-	-	+	+ with
May be produced by patient's state, etc	+	+	+	- clinical
Known response pattern to suspected drug	-	-	+	+
Disappears or decreases on Stopping or reduction in dose	-	-	-	+
Reappears on rechallenge	-	-	-	+

D2. Adverse event pattern of events

a. Single event: The event occurred just once, and has ended at the time of reporting.
b. Continuous: The event began just once, and is still ongoing at the time of reporting.
c. Intermittent: The event has gone through at least one cycle of starting, stopping, and starting again.

D3. Adverse event status

a. Resolved, no residual effect: after adverse event ends, the patient returns to pre-adverse event status.
b. Resolved with sequelae: After adverse event ends, the patient does not return to pre-adverse status.
c. Continuing: The adverse event is still ongoing at the time of reporting.
d. Disability: defined by the FDA as "substantial disruption of a person's ability to conduct normal life functions.
e. Death
f. Medically stable according to Principal Investigator
g. Followed by physician outside of HALT-C Trial

D4. Peginterferon alfa-2a Dose Adjustment Guidelines

Specific dose adjustment guidelines for peginterferon alfa-2a are provided in the tables below for post-treatment elevated serum ALT activities, neutropenia, and thrombocytopenia. For other adverse effects considered to be possibly related to peginterferon alfa-2a, including laboratory abnormalities, adverse events, and vital signs changes, investigators should utilize the table below labeled "General Dose Reduction Guidelines." When practicable, abnormal laboratory results should be confirmed as soon as possible following notification of the investigator. If appropriate, downward adjustments in one level increments (see section M.1.) should be considered. The lowest dose of peginterferon alfa-2a that should be administered is 45 μg. It should be kept in mind that whereas these guidelines should be generally

followed to promote consistency across centers, other responses by an investigator may be more appropriate in some circumstances.

a. General Dose Reduction Guidelines

Number of Dose Reduction Levels (see Section M.1.b.)					
Mild	Moderate Limited	Moderate Persistent	Severe Limited	Severe Persistent	Life-Threatening
0	0	0 – 1	0 - 1	1 – 2	Stop Drug

b. Dose Adjustments for Low Absolute Neutrophil and Platelet Counts for patients who enter the trial with ANC > 1500 and Platelet Count > 75,000.

Parameter	Downward Dose Adjustment
ANC (cells/mm^3)	
≥1000	None
750 - 999	Week 1 - 2*: Immediate 1 Level adjustment<br>Week 3 and greater: None
500 - 749	Week 1 - 2: Delay or hold dose until ≥750 then resume dose with 1 Level adjustment<br>Week 3 and greater: Immediate 1 Level adjustment
250 - 499	Week 1 - 2: Delay or hold dose until ≥750 then resume dose with 2 Level adjustment<br>Week 3 and greater: Delay or hold dose until ≥750 then resume dose with 1 Level adjustment
<250	Stop Drug
Platelet Count (cells/mm^3)	
≥50,000	None
35,000 - 49,000	Delay or hold dose until ≥50,000 then resume dose with 1 Level adjustment
25,000 - 34,000	Delay or hold dose until ≥50,000 then resume dose with 2 Level adjustment
<25,000	Stop Drug

*Week 1-2: Signifies the abnormality was noted within the first 2 weeks of the initiation of test drug treatment.

**Week 3 and greater: Signifies the abnormality was noted more than 2 weeks following the initiation of test drug treatment.

c. Dose Adjustments for Low Absolute Neutrophil and Platelet Counts for patients who enter the trial with neutrophils between 1,000/mm^3 to 1,500/mm^3 and Platelet Count from 50,000/mm^3 up to 75,000/mm^3.

Platelet Count (cells/mm^3)	Downward Dose Adjustment
≥40,000	None
20,000 - 40,000	Delay or hold dose for 1 week until ≥40,000 then resume dose with 1 Level adjustment
<20,000	Stop Drug

ANC (cells/mm^3)	Downward Dose Adjustment
≥750	None
250 - 750	Delay or hold dose until ≥750 then resume dose with 1 Level adjustment
<250	Stop Drug

Safety measures for patients with platelet count under 75,000/mm^3 and/or neutrophils under1,500/mm^3 :

1. The ribavirin should be unchanged but may be lowered at the PI's discretion. Patients will start at a reduced dose of 90 μg of peginterferon alfa-2a once weekly.
2. Patients will be monitored more closely by adding a CBC with differential blood tests at Week 1 and Week 6.
3. Patients will be asked to hold weekly dose of peginterferon alfa-2a until the results of the CBC with differential blood test are assessed by the PI.
4. Patients entering Lead-in with a lowered platelet and/or neutrophil count will have ongoing monitoring by the DSMB for the first 8 weeks of treatment.
5. A separate dose reduction scheme will be followed for these patients as indicated in Table c above.
6. Dose adjustment upwards is at the discretion of the PI after 8 weeks in the Lead-in.

d. Dose Adjustments for Elevated Serum ALT (see note below)

Baseline Serum [ALT]	On-Treatment Serum [ALT]	Downward Dose Adjustment
≤100	<200	None
	200 – 300	Repeat test in 1 week. If ALT decreased or stable (≤10% increase), continue at present dose and follow every 1-2 weeks to assure stability. If increased by >10%, decrease dose by 1 Level and follow with weekly testing until ALT is stable or decreased.
	301 – 500	Repeat test prior to administering dose. If ALT decreased or stable (≤10% increase), decrease by 1 Level and follow weekly to assure stability. If increased by >10%, hold dose until ALT decreases to <300 then resume test drug at 2 Level decrease and follow every 1-2 weeks until stable. If a further 10% increase occurs, stop test drug.
	>500	Hold test drug until ALT decreased to <300 then resume test drug at 2 Level decrease and follow every 1-2 weeks. If ALT >300, stop test drug.
101 - 200	≤300	None
	301 – 500	Repeat test prior to administering dose. If ALT decreased or stable (≤10% increase), decrease by 1 Level and follow weekly to assure stability. If increased by >10%, hold dose until ALT decreases to <300 then resume test drug at 2 Level decrease and follow every 1-2 weeks until stable. If a further 10% increase occurs, stop test drug.
	>500	Hold test drug until ALT decreased to <300 then resume test drug at 2 Level decrease and follow every 1-2 weeks. If ALT >300, stop test drug.
201 – 300	≤400	None
	401 – 500	Repeat test prior to administering dose. If ALT decreased or stable (≤10% increase), decrease by 1 Level and follow weekly to assure stability. If increased by >10%, hold dose until ALT decreases to <300 then resume test drug at 2 Level decrease and follow every 1-2 weeks until stable. If a further 10% increase occurs, stop test drug.

Baseline Serum [ALT]	On-Treatment Serum [ALT]	Downward Dose Adjustment
	>500	Hold test drug until ALT decreased to <300 then resume test drug at 2 Level decrease and follow every 1-2 weeks. If ALT >300, stop test drug.
301 - 500	≤500<br><br>>500	None<br><br>Repeat test prior to administering dose. <u>If ALT decreased or stable (≤10% increase)</u>, decrease by 1 Level and follow weekly to assure stability. <u>If increased by >10%</u>, hold dose until ALT decreases to less than baseline then resume test drug at 2 Level decrease and follow every 1-2 weeks until stable. If a further 10% increase occurs, stop test drug.
> 500	≤25% Increase<br>>25% Increase	None<br><br>Repeat test prior to administering dose. <u>If ALT decreased or stable (≤10% increase)</u>, decrease by 1 Level and follow weekly to assure stability. <u>If increased by >10%</u>, hold dose until ALT decreases to less than baseline then resume test drug at 2 Level decrease and follow every 1-2 weeks until stable. If a further 10% increase occurs, stop test drug.

Note: This table is based on an upper limit of normal (ULN) serum ALT of 43 U/L for men and 34 U/L for women. The ULN at the investigator's laboratory should be considered when employing this table.

E. Screening Consent Form

F. Trial Consent Form
